# Elucidating the Near-Infrared Photoluminescence Mechanism of Homometal and Doped M_25_(SR)_18_ Nanoclusters

**DOI:** 10.1021/jacs.3c06543

**Published:** 2023-08-29

**Authors:** Zhongyu Liu, Meng Zhou, Lianshun Luo, Yitong Wang, Ellen Kahng, Rongchao Jin

**Affiliations:** Department of Chemistry, Carnegie Mellon University, Pittsburgh, Pennsylvania 15213, United States

## Abstract

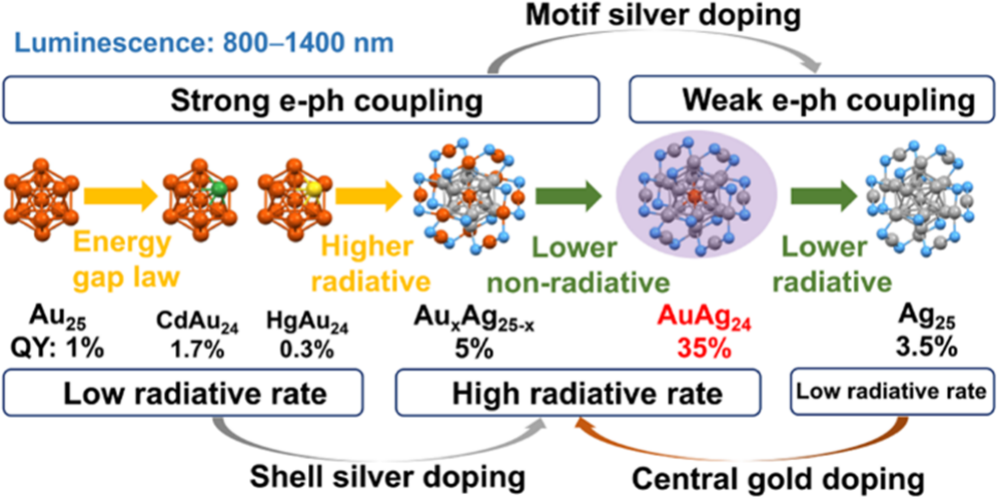

More than a decade of research on the photoluminescence (PL) of classic Au_25_(SR)_18_ and its doped nanoclusters (NCs) still leaves many fundamental questions unanswered due to the complex electron dynamics. Here, we revisit the homogold Au_25_ (ligands omitted hereafter) and doped NCs, as well as the Ag_25_ and doped ones, for a comparative study to disentangle the influencing factors and elucidate the PL mechanism. We find that the strong electron–vibration coupling in Au_25_ leads to weak PL in the near-infrared region (∼1000 nm, quantum yield QY = 1% in solution at room temperature). Heteroatom doping of Au_25_ with a single Cd or Hg atom strengthens the coupling of the exciton with staple vibrations but reduces the coupling with the core breathing and quadrupolar modes. The QYs of the three MAu_24_ NCs (M = Hg, Au, and Cd) follow a linear relation with their PL lifetimes, suggesting a mechanism of suppressed nonradiative decay in PL enhancement. In contrast, the weaker electron–vibration coupling in Ag_25_ leads to higher PL (QY = 3.5%), and single Au atom doping further leads to a 5× enhancement of the radiative rate and a suppression of nonradiative decay rate (i.e., twice the PL lifetime of Ag_25_) in AuAg_24_ (hence, QY 35%), but doping more Au atoms results in gold distribution to staple motifs and thus triggering of strong electron–vibration coupling as in the MAu_24_ NCs, hence, counteracting the radiative enhancement effect and giving rise to only 5% QY for Au_*x*_Ag_25–*x*_ (*x* = 3–10). The obtained insights will provide guidance for the design of metal NCs with high PL for lighting, sensing, and optoelectronic applications.

## Introduction

Photoluminescence (PL) is one of the most important properties of atomically precise metal nanoclusters (NCs). The research is driven by the motivation of understanding the PL mechanism and also by the promising applications of luminescent NCs in fields such as biomedical imaging/sensing, solar concentrators, and lighting.^1−3^ However, the PLQY of most metal NCs is quite low (i.e., PLQY < 1%); thus, PL enhancement is of critical importance. Several effective strategies have been reported, including aggregation-induced emission,^3^ ligand engineering,^4−8^ tailoring of the staple motifs,^9−11^ and heteroatom doping into the core.^12−15^ Nevertheless, the detailed PL mechanism has not been fully elucidated in many cases.

Among the thiolate (−SR)-protected metal NCs, the icosahedral [M_25_(SR)_18_]^−^ NCs (M = Au, Ag) often serve as the model systems for investigating the physical and chemical properties of metal NCs because of their easy syntheses and superatomic closed shell electron configuration (1S^2^|1P^6^).^16^ A full understanding of the excited-state relaxation in [M_25_(SR)_18_]^−^ NCs can provide guidance for the fundamental studies of other NCs toward the tailoring of their optical functionality, but this is still hampered by the complex electronic dynamics^16^ in such NCs caused by the intricate interactions between the kernel and the surface staple motifs.^4^

The structure of [Au_25_(SR)_18_]^−^ NC was solved as early as 2008 with phenylethanethiolate (abbrev. PET) as the protecting ligand.^17,18^ Single-crystal X-ray diffraction revealed that [Au_25_(PET)_18_]^−^ (denoted Au_25_ hereafter) consists of an icosahedral Au_13_ kernel (or core) and six Au_2_(PET)_3_ staple motifs on the surface. The study on the structure–PL correlation of Au_25_ has been carried out ever since its structure was determined,^7^ but the full emission spectrum was not obtained for a long period. With a broadband detector that covers both the visible and near-infrared range, Liu et al. recently determined the full emission profile of Au_25_ to be a single broad peak centered at around 1100 nm with a PLQY of 1%.^4^ For the silver counterpart (i.e., [Ag_25_(SR)_18_]^−^), its structure was reported in 2015 and also comprises an icosahedral Ag_13_ kernel and six Ag_2_(SR)_3_ staple motifs,^19^ being similar to Au_25_ but with slight differences in bond lengths and bond angles. For [Ag_25_(SR)_18_]^−^, 2,4-dimethylbenzenethiol (2,4-DMBT) is the most commonly used ligand and hereafter [Ag_25_(2,4-DMBT)_18_]^−^ is denoted as Ag_25_. The emission peak of Ag_25_ is located at 970 nm, and both Au_25_ and Ag_25_ exhibit a large Stokes shift (∼0.5 eV), indicating structural changes in the excited state.^16^ Although much effort has been devoted to revealing the PL mechanism of the Au_25_ NC,^6,16^ a universal picture that can rationalize all of the experimental results is still lacking; similarly, the case of Ag_25_ also requires more elucidation. Meanwhile, their low PLQY (<5%) makes the study of PL mechanisms quite challenging, especially in time-resolved PL analyses.

In regard to PL enhancement,^3−15^ heteroatom doping is a facile and versatile strategy to tailor the optical and electronic properties of the NCs.^9,12−15^ For the heteroatom doping into Au_25_, the structures of Pd, Pt, Cd, Hg, and Ag-doped M_25_ NCs (where M = metal) have been successfully solved, and the preferred doping sites were found to depend on the group of the doping atoms (e.g., Pd/Pt versus Cd/Hg).^20−23^ Similarly, in the case of Ag_25_, the structures of Pd-, Pt-, and Au-doped M_25_ NCs have also been successfully determined.^19,24,25^ The PL properties of these M_25_ nanoclusters have drawn great attention, but the understanding of the PL mechanism is still limited due to the very complex excited-state dynamics. In addition, because of the limitations of PL instruments (e.g., the cutoff of the near-infrared part of the emission by visible range detectors), the reported emission spectra of many M_25_ NCs may not be complete, which results in some deficient conclusions.

Herein, we select two series of M_25_ NCs to study the PL mechanism, (i) the Au_25_-based series (including Au_25_, CdAu_24_, and HgAu_24_) and (ii) the Ag_25_-based series (including Ag_25_, AuAg_24_, and Au_*x*_Ag_25–*x*_). To make a better comparison with the parent [Au_25_(SR)_18_]^−^ and [Ag_25_(SR)_18_]^−^, all of the doped NCs are chosen to have a closed shell of 8-electron configuration; thus, the Pd and Pt doping cases are not included since they typically lead to 6e systems. We performed temperature-dependent steady-state and time-resolved PL measurements to understand how the doping atoms affect both the radiative and nonradiative decays. The mono-heteroatom (Cd or Hg) doping into Au_25_ is found to significantly affect the electron–acoustic phonon interaction (i.e., the nonradiative decay), while gold atom doping into Ag_25_ significantly affects both radiative relaxation and nonradiative decay processes. The obtained insights not only provide some guiding principles for the design of NCs with high photoluminescence but also will promote the research on the electroluminescence (in light-emitting diodes^3^) and electrochemiluminescence as well as solar concentration applications^2^ of the NCs.

## Results and Discussions

### Optical Characterization of MAu_24_ (M = Cd, Hg, and Au) NCs

For the Au_25_-based series of NCs, Au_25_(PET)_18_^–^ was synthesized following a previously developed one-pot synthesis method.^26^ The Cd or Hg monodoped NCs (denoted as CdAu_24_ and HgAu_24_) were synthesized by a reaction between Au_25_(PET)_18_^–^ and metal thiolate complexes (i.e., Cd(PET)_2_, Hg(PET)_2_).^22,27^ Thin-layer chromatography (TLC) was employed to separate CdAu_24_ and HgAu_24_ from the crude products. Crystals of CdAu_24_ and HgAu_24_ were obtained by diffusion of acetonitrile into toluene solutions of the NCs. All measurements used the crystallized NCs that were redissolved in solvents in order to ensure the highest purity of the NCs.

**Figure 1:**
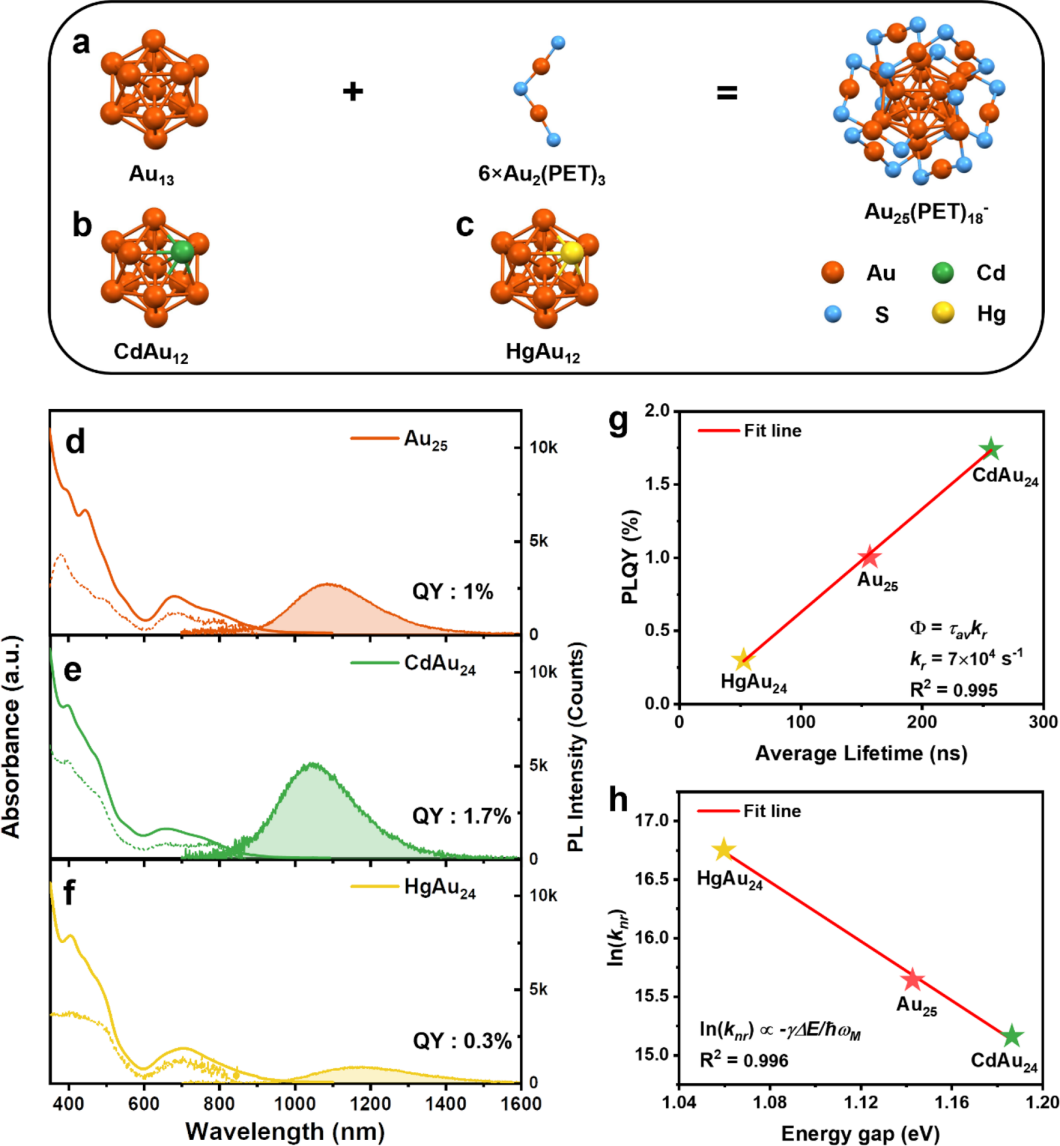
(a) Atomic structure of Au_25_ (carbon tails and Au(core)–Au(shell) bonds are omitted for clearity). (b) Structure of the CdAu_12_ core. (c) Structure of the HgAu_12_ core. The structures of the NCs were redrawn from the CIF.^27−30^ Absorption (solid line) and PL (shadowed area) spectra of (d) Au_25_, (e) CdAu_24_, and (f) HgAu_24_ in deaerated CDCl_3_ (with N_2_). Dashed lines represent the excitation spectra of PL at (d) 1085 nm, (e) 1045 nm, and (f) 1170 nm. (For PL measurements: excitation at 500 nm with 0.2 OD, slit width 8 nm, and emission slit 8 nm.) (g) Plot of PLQY versus average lifetime for the three gold-based NCs. The red line is a linear fit to Φ = τ_av_*k*_r_Φ_isc_ (assume Φ_isc_ = 1). (h) Plot of ln(*k*_nr_) versus the photoluminescence gap for the three NCs. The red line is a linear fit to the energy gap law.

Based on the reported single-crystal X-ray diffraction (SCXRD) results, the structure of Au_25_ comprises a Au_13_ icosahedral core and six Au_2_(PET)_3_ staple motifs^17^ (Figure 1a). Due to the insufficient difference of atomic mass between Hg/Cd and Au atoms, it is not easy to reliably determine the Cd or Hg doping site by SCXRD. The Hg or Cd atom was earlier reported to replace one Au atom on the Au_2_(PET)_3_ staple motif or the central gold in the icosahedron.^28−30^ However, recent NMR results and theoretical simulations proved that the actual doping site of the Hg or Cd atom is on the Au_12_ icosahedral shell, resulting in an Au@MAu_11_ kernel (Au@MAu_11_ is denoted as MAu_12_ below, M = Au, Cd, or Hg, Figure 1a–c).^27,31^ The difference in the six staple motifs of the three NCs is negligible since all are gold-containing Au_2_(PET)_3_ staples.

Here, we use deuterated chloroform (CDCl_3_) as the solvent for all the room-temperature optical measurements in the near-infrared (NIR) region. Compared to other solvents, CDCl_3_ has weaker NIR absorption (i.e., vibrational overtones), which can alleviate the solvent absorption-induced distortion of the NIR PL spectra. Figure 1d–f (solid lines) shows the room-temperature optical absorption spectra of MAu_24_ in CDCl_3_ (M = Au, Cd, or Hg). The characteristic peak of Au_25_ at 678 nm is slightly shifted to 657 nm in CdAu_24_ and 703 nm in HgAu_24_. The ∼800 nm shoulder peak of Au_25_ experienced a blue shift to ∼760 nm in CdAu_24_ but vanished in HgAu_24_, suggesting that the single heteroatom affects the energy levels and the electronic transitions. The PL spectra of MAu_24_ NCs are measured in deaerated CDCl_3_ (with N_2_) under 500 nm excitation (Figure 1d–f, shaded areas). On a note, the existence of O_2_ or higher excitation energy could potentially oxidize or damage the NCs and result in a side-product with emission at around 800 nm. All three NCs exhibit a single emission peak in the range of visible to NIR (detector’s range: 500–1700 nm). The emission peak of Au_25_ is centered at 1080 nm, whereas CdAu_24_ and HgAu_24_ are at 1045 nm and 1170 nm, respectively. A comparison of PL spectra in DCM and CDCl_3_ is shown in Figure S1, demonstrating a negligible solvent effect. The PL excitation spectra (Figure 1d–f, dashed lines) of all three NCs match well with their UV–vis absorption peaks, suggesting that the emission originates from the first excited state after the hot exciton relaxes.

The photoluminescence quantum yield (PLQY) of Au_25_ was previously determined to be 1% via a relative method using the IR-1061 dye as the standard.^4^ In this work, the PLQY of Au_25_ is further confirmed using the PLQY of AuAg_24_(2,4-DMBT)_18_^–^ as the standard. The PLQYs of CdAu_24_ and HgAu_24_ were obtained by comparing their integrated peak areas with that of Au_25_ and were determined to be 1.7 and 0.3%, respectively. The PL lifetimes were measured by a multichannel scaling (MCS) single-photon counting technique. As shown in Figure S2, the decay curves of all three NCs can be well fitted by monoexponential functions. The fitting results suggest that the PL lifetime is 53 ns for HgAu_24_, 155 ns for Au_25_, and 257 ns for CdAu_24_. The measured lifetimes are consistent with the previous results extracted from the transient absorption spectroscopy (TAS) analyses.^32^

The nature of the PL of Au_25_ has been discussed for a long time, but it is still under debate. The existence of a triplet state in Au_25_ was earlier proved by singlet oxygen generation experiments conducted by Kawasaki et al.^33^ In later work, Agrachev et al.^34^ also confirmed that all of the MAu_24_ NCs (M = Au, Cd or Hg) can be singlet oxygen sensitizers, and their activity was found to be related to their excited-state lifetimes (determined by TAS).^32^ The NIR emissions of the three NCs have the same lifetimes as the TAS results,^32^ suggesting that the transient absorption and PL decay involve the same excited state. Since the longest component in the TAS comes from the triplet state, the ∼1100 nm emission should arise from the same triplet state. Therefore, we can ascribe the emission peak at ∼1100 nm to the phosphorescence from the MAu_12_ kernel. The lifetime of MAu_24_ (i.e., ranging from 53 to 257 ns) is much shorter than the typical lifetime of phosphorescence (i.e., microseconds), but similar results have been observed in metal–organic complexes.^35^ In TAS analyses, a picosecond-scale lifetime (2–5 ps) was observed in all three MAu_24_ NCs, which is similar to the picosecond lifetime in the charge-neutral Au_25_.^32,36^ Previously, this picosecond lifetime was assigned to the transition between the excited state and the surface trap state. Now, we believe it is better to be assigned as the lifetime of the S_1_ state.

Interestingly, we found that the PLQYs of the three NCs are proportional to their PL lifetime (Figure 1g). Theoretically, the PLQY of phosphorescence (Φ_p_) is defined as
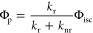
1where Φ_isc_ is the quantum yield of intersystem crossing (ISC), *k*_r_ is the radiative rate, and *k*_nr_ is the nonradiative rate. In principle, the PLQY can be enhanced by accelerating the radiative decay (i.e., large *k*_r_) and/or suppressing the nonradiative decay (small *k*_nr_). To reveal the mechanism, eq 1 is rewritten as
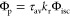
2where τ_av_ is the measured PL lifetime. To simplify the calculation, we assume that Φ_isc_ is close to unity, which is justifiable.^13^ Then, the linear relationship between the PLQY and the lifetime of the three NCs indicates that the radiative rates (*k*_r_) of the three NCs are approximately the same. Following this logic, a linear fitting is given in Figure 1g and the common *k*_r_ of the three NCs is determined to be (7 ± 0.1) × 10^4^ s^–1^. This radiative rate is close to the rate constant of phosphorescence in Au_13_ NCs,^13,14^ which further supports the phosphorescence nature in the MAu_24_ NCs. Given the above results, we can conclude that the radiative rates of these three NCs are essentially the same; thus, the doping atom only exerts its effect through the nonradiative channel, which accordingly affects the observed PLQY.

Another interesting relationship is that the nonradiative rates of the three NCs are found to follow the energy gap law (Figure 1h). The energy gap law is widely applied to understand the multiphonon-assisted emission mechanism.^37^ The formula of the simplest version of the energy gap law^38^ is given in eq 3
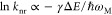
3where ln(*k*_nr_) is the natural logarithm of the nonradiative rate constant, γ is a molecular parameter, *ℏω*_M_ is the highest energy of phonon that assists the nonradiative decay, and Δ*E* is the energy gap. Of note, here we take the PL peak position as the energy gap. Considering the similarity in the geometrical structure of these three NCs as well as their electronic structure, the molecular parameter γ can be treated as the same for the three NCs. Then, it means that the ℏω_*M*_ is roughly consistent in all three NCs. The main phonon that affects the electronic transition in Au_25_ NCs in the solution phase was determined as the vibration from the Au_2_(SR)_3_ staple motifs.^39^ Since the motifs of these three NCs are the same, it is reasonable to observe such a linear relationship.

### Temperature-Dependent PL Analysis of MAu_24_ (M = Cd, Hg, and Au) NCs

Since the nonradiative relaxation through vibrations of staple motifs is strong for all three NCs and may hinder the understanding of doping effects on the core vibration, we carried out temperature-dependent PL measurements from room temperature down to 20 K (liquid helium as the cryogen). For such measurements, MAu_24_ NCs were embedded in a polystyrene film matrix. Our previous work demonstrated that the vibrations from staple motifs could be suppressed in thin films, which allowed us to obtain insights into the core vibrations pertaining to the MAu_12_ icosahedron.^4,39^ In cryogenic experiments, the chamber of the cryostat was filled with helium gas during the measurements to avoid interference from oxygen.

At room temperature, as shown in Figure S3a–c, the emission profiles of all three NCs in polystyrene thin films are consistent with their solution counterparts but with higher intensity due to the suppression of the motif vibration. Specifically, the PLQY of Au_25_ increases to 1.8%, whereas CdAu_24_ and HgAu_24_ increase to 7 and 2.2%, respectively (Figure S3a–c); generally, the enhancement is a few times greater. The lifetimes of all three NCs become prolonged when embedded in polystyrene thin films (Figure S3d–f).

**Figure 2:**
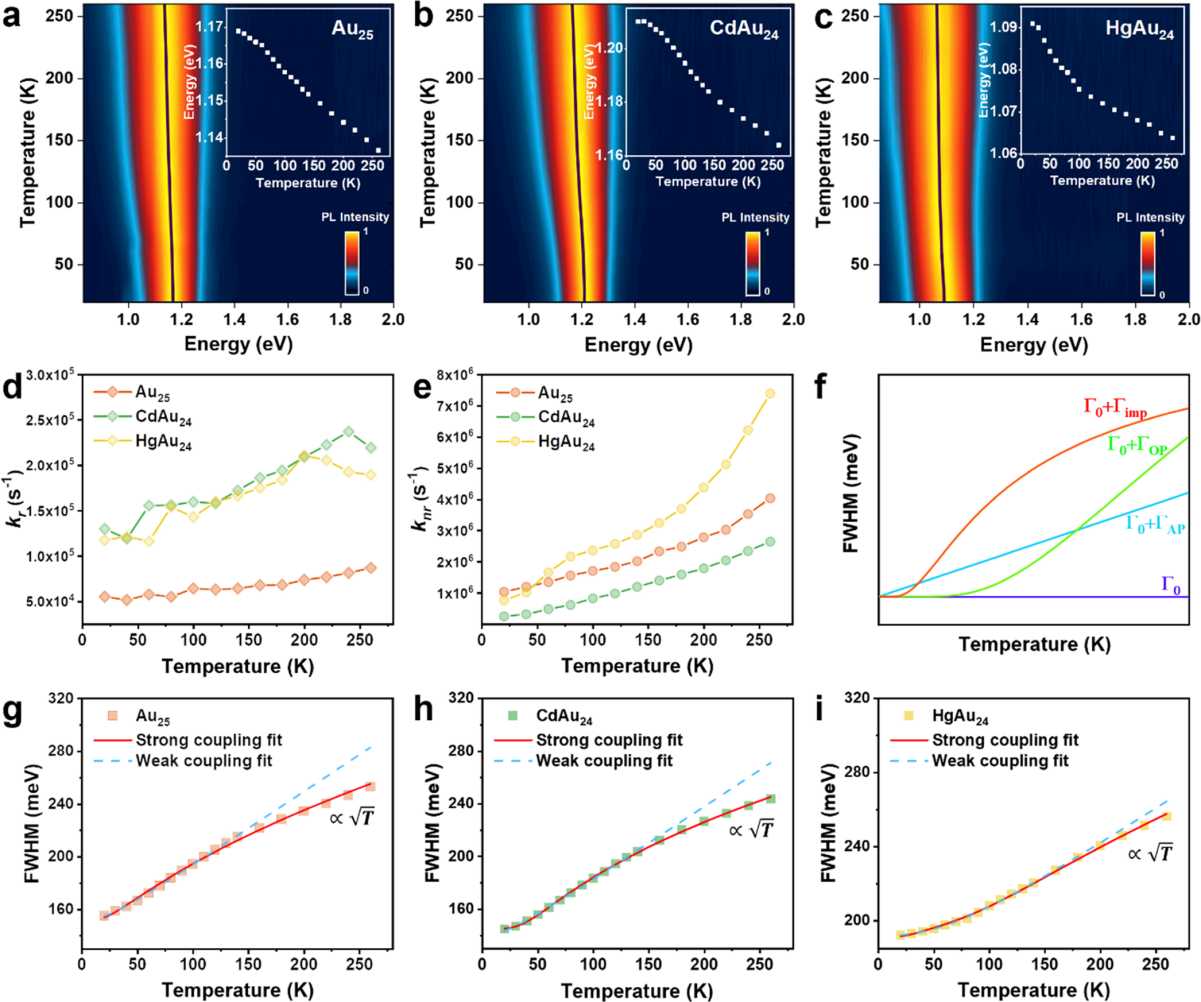
Temperature-dependent PL spectra of (a) Au_25_, (b) CdAu_24_, and (c) HgAu_24_ with excitation at 500 nm. (d) Temperature-dependent radiative rates of Au_25_, CdAu_24_, and HgAu_24_. (e) Temperature-dependent nonradiative rates of Au_25_, CdAu_24_, and HgAu_24_. (f) Functional form of the temperature dependence of the contributions to the PL linewidth. The FWHM of the steady-state PL spectra as a function of temperature for (g) Au_25_, (h) CdAu_24_, and (i) HgAu_24_. The blue dashed lines fit the results to eq 4, and the red lines fit the results to eq 5 (see main text).

The temperature-dependent PL spectra of the three NCs are given in Figure 2a–c, where the black traces and white insets represent the temperature-dependent peak positions. The emission peaks of all three samples become narrower and show a gradual blue shift as the temperature decreases. A monotonic increase of PL intensity (Figure S4a–c) is observed for all three MAu_24_ NCs with temperature decreasing from room temperature to 20 K. Nevertheless, even with the suppression of vibrations, the PLQY for the three samples is still below 30% at 20 K (Figure S4a–c). The low-temperature PLE spectra (Figure S4d–f) are consistent with the previously reported low-temperature absorption spectra,^32^ suggesting that the emission peak is only related to the first excited state (S_1_) at different temperatures. The temperature-dependent lifetimes (Table S1) show a monotonic increase with decreasing temperature. Moreover, the lifetimes for the three NCs at 20 K all reach the microsecond scale, which further supports the phosphorescence nature of the observed NIR emission.

As shown in Figure 2d, all three NCs show higher radiative rates in the thin film (*k*_r_ ranging from 9 × 10^4^ to 2 × 10^5^ at r.t.) than in the solution (*k*_r_ = 7 × 10^4^ s^–1^ at r.t.). The enhancement of the radiative rate in the solid state may originate from aggregation-induced slight overlapping of wavefunctions in the solid state, which may boost the transition from the T_1_ to the S_0_ state. Interestingly, the radiative rate of CdAu_24_ and HgAu_24_ in thin films are close at all temperatures (Figure 2d), and both also surpass that of Au_25_. Such a difference may originate from the different charge states of MAu_24_ NCs, among which CdAu_24_ and HgAu_24_ are charge neutral, while Au_25_ possesses a negative charge, which may result in electrostatic repulsion between NCs in the solid state, hence, less aggregation-induced enhancement in the case of Au_25_. Meanwhile, a decrease in radiative rate with temperature decrease is observed for all three NCs, suggesting activation energy is present for radiative recombination.^40^ Overall, the nonradiative rates (Figure 2e, i.e., of order 10^6^) are found to be roughly an order of magnitude larger than the radiative rates. The *k*_nr_ trend is HgAu_24_ > Au_25_ > CdAu_24_ when the temperature is above 80 K, but the *k*_nr_ of HgAu_24_ becomes smaller than that of Au_25_ when the temperature is lower than 80 K. The monotonic decrease of *k*_nr_ as the temperature decreases is because of the suppression of the phonon population at low temperatures. The rapid nonradiative recombination (i.e., of order 10^6^) is the main reason for the low PLQY of these three NCs.

To understand the origin of rapid nonradiative rates in three gold NCs, we carried out an analysis of temperature-dependent emission broadening. Such an analysis has been applied to understand the electron–phonon interactions, which is the main nonradiative relaxation pathway in semiconductor materials.^41−43^ The linewidth (Γ) is represented by the full width at half maximum (FWHM). For most semiconductor materials, the temperature-dependent PL broadening follows a weak electron–phonon coupling (e–ph) and it can be described by eq 4 below.^43,44^ In this weak regime, the product of the phonon field fluctuation amplitude (Δ) and fluctuation correlation time (τ_c_) is smaller than ℏ, which typically results in a Lorentzian line shape at low temperatures.^45,46^
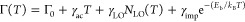
4In the equation, Γ_0_ is the temperature-independent intrinsic linewidth of materials, γ_ac_ and γ_LO_ are the coupling coefficients of an electron with acoustic phonon (ac) and longitudinal optical (LO) phonon, respectively, and *N*_LO_(*T*) is the Bose–Einstein distribution () that describes the temperature-dependent population of phonon modes. The last term in eq 4 accounts for the contribution from ionized impurities. The temperature-dependent general trends of the four contributors are plotted in Figure 2f.

In the case of gold NCs, their formulas are precisely defined, and the samples are molecularly pure; thus, the contribution from impurities is irrelevant and can be ignored. It is worth noting that *N*_LO_(*T*) can be regarded as ∝*T* when the temperature is high (i.e., *T* > 100 K, Taylor expansion), suggesting that the sum over the remaining three contributors should result in a linear increase of FWHM with increasing *T* (when *T* > 100 K). The FWHM of the three NCs at different temperatures are extracted and plotted in Figure 2g–i. Interestingly, the temperature-dependent trends of all three NCs in the high-temperature region do not follow the expected linear relationship, suggesting the common weak e–ph coupling approximation (Figure 2g–i, blue dashed lines) is not applicable here.

In contrast to the linear relationship (∝*T*) for the *weak* coupling, Toyozawa theoretically demonstrated that the temperature-dependent evolution of the excitonic linewidth would be proportional to  under strong electron–phonon coupling, in which the linewidth broadening can be described as eq 5(45−47)
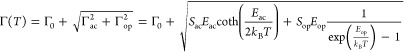
5where Γ_0_ is the temperature-independent intrinsic linewidth, *S*_ac_ and *S*_op_ are the coupling strengths for acoustic phonons and optical phonons, respectively, and *E*_ac_ and *E*_op_ are the average energy of acoustic phonons and optical phonons. The red lines in Figure 2g–i show the fitting results based on eq 5. Apparently, the fitting by the strong coupling model is much better than the weak coupling model. Therefore, strong electron–phonon interactions are involved in the MAu_24_ NCs.

The extracted parameters by fitting with the strong e–ph coupling model are listed in Table 1. As afore-discussed, the low-frequency acoustic vibrations in Au_25_ are from the breathing mode and quadrupolar-like mode of the Au_13_ kernel, while the high energy optical phonon modes are from the vibrations of Au_2_(SR)_3_ staple motifs.^4,48−50^ The average energy *E*_ac_ of acoustic phonon modes for the three NCs are quite close (Table 1), suggesting that the doped heteroatoms have a trivial contribution to the periodical expansions/contractions of the MAu_12_ kernel. Nevertheless, the electron–acoustic-phonon coupling strength *S*_ac_ in Au_25_ is found to be significantly stronger than the Cd- and Hg-doped MAu_24_. It means that heteroatom doping could weaken the exciton-vibration coupling within the core. Yu et al. theoretically simulated the Raman spectra of Cd- and Hg-doped MAu_24_ NCs and found that the intensity of the low-frequency vibration (i.e., < 100 cm^–1^) is very low and much weaker than Pd- or Pt-doped MAu_24_ NCs.^50^ Meanwhile, Krishnadas et al. experimentally measured the Raman spectra of Au_25_ and found strong signals in the low-frequency region (<100 cm^–1^).^51^ Considering these two facts, it is reasonable for us to observe less coupling after Cd and Hg doping. The breathing mode is known as the main acoustic vibration in Au_25_ and it is highly symmetric.^4,36,48^ The doping of Cd or Hg atom into Au_25_ breaks the symmetry of the core due to the asymmetric distribution of electron density.^22,50^ Therefore, such a dissymmetry diminishes the scalar product of the breathing mode atoms’ displacement and excitation profile integrated over the spherical volume,^48^ which weakens the coupling between the exciton and acoustic phonon after Cd or Hg doping.

**Table 1:** Extracted Parameters for the Three MAu_24_ NCs (Fitted by eq 5)

	Γ_0_, meV	*S*_ac_, meV	*E*_ac_, meV	*S*_op_, meV	*E*_op_, meV
Au_25_	109	393	4	423	35
CdAu_24_	137	45	5	757	18
HgAu_24_	181	69	7	584	40

As shown in Table 1, the average energy of optical phonon *E*_op_ in Au_25_ and HgAu_24_ are determined to be ∼40 meV (∼300 cm^–1^), which is consistent with previous cryogenic absorption measurements.^4^ The coupling strength of the optical phonon *S*_op_ is high for all three NCs (Table 1), demonstrating that the coupling of the exciton with the optical phonon modes is strong. Due to the high energy of the optical phonon, their population is very small at low temperatures (i.e., <100 K); thus, the acoustic phonon becomes the main contributor to nonradiative relaxation. Since CdAu_24_ and HgAu_24_ have weaker coupling to an acoustic phonon, these two NCs should have smaller nonradiative rates (compared to Au_25_) when the temperature is low, which is consistent with the results in Figure 2e. In contrast, at room temperature, the doping of the heteroatom suppresses the electron–acoustic-phonon coupling, but the scattering of electrons by optical phonons is extremely strong for all three NCs, which inevitably results in low PLQY.

**Scheme 1:**
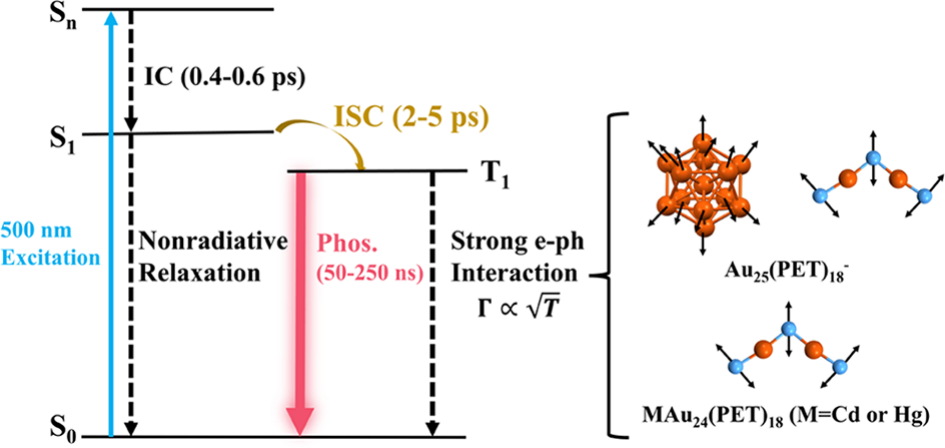
Emission Mechanism for the MAu_24_ (M = Au, Cd, and Hg) NCs

Combining the discussions above, we summarize the mechanism in Scheme 1. Briefly, the ∼1100 nm NIR emission from the MAu_24_ NCs is ascribed to phosphorescence based on the following facts: (1) all three MAu_24_ NCs can serve as singlet oxygen sensitizers, suggesting the existence of triplet-state populations; (2) the time-resolved PL measurements showed a single lifetime for their emission; and (3) the lifetimes determined from time-resolved PL measurements are consistent with the longest lifetime component in previous transient absorption results. Additionally, earlier TAS experiments demonstrated the presence of a picosecond-scale lifetime at 730 nm excitation,^32^ suggesting that this lifetime originates from the lowest excited state. Therefore, we assign the picosecond lifetime to the decay of the S_1_ state (i.e., intersystem crossing and vibrational relaxation). Moreover, an unusually strong electron–phonon interaction is found in all three NCs. Both acoustic phonons and optical phonons contribute to the electron–phonon scattering in Au_25_ as indicated by large *S*_ac_ and *S*_op_ values, while the acoustic phonon contribution is suppressed in CdAu_24_ and HgAu_24_ due to asymmetric doping. The strong electron–phonon interactions result in fast nonradiative relaxation, hence, low PLQY in the three NCs. The fast nonradiative relaxation is also the reason for the complete suppression of fluorescence and the unusually short lifetime (50–257 ns) of phosphorescence at room temperature, as dictated by the relation . One may question why the strong electron–phonon interaction is not able to suppress the phosphorescence completely; this is because the nonradiative relaxation of the triplet state also needs to go through a slow spin-flipping step, which slows down the entire nonradiative process. Overall, the monoatom doping could significantly affect the electron–acoustic phonon coupling but will not promote the PLQY a lot because the nonradiative relaxation by optical phonons (i.e., motif vibrations) is extremely strong in MAu_24_ NCs.

### Insight from the Silver Series of Ag_25_, AuAg_24_, and Au_*x*_Ag_25–*x*_ (*x* = 3–10) NCs

To further understand the metal-doping effects, we investigated a second system related to Au_25_ and doped NCs, that is, Ag_25_ and doped NCs, collectively as Au_*x*_Ag_25–*x*_(2,4-DMBT)_18_^–^ NCs (with the number of gold atoms *x* = 0, 1, and 3–10). Previous SCXRD results showed that all these silver-based NCs possess an M_13_ kernel and six M_2_(SR)_3_ staple motifs, being the same as the structure of Au_25_.^19,24,52^ Therefore, these silver-based NCs can also be viewed as heavily silver-doped gold NCs, which further enable us to understand the multiatom doping effects in Au_25_.

**Figure 3:**
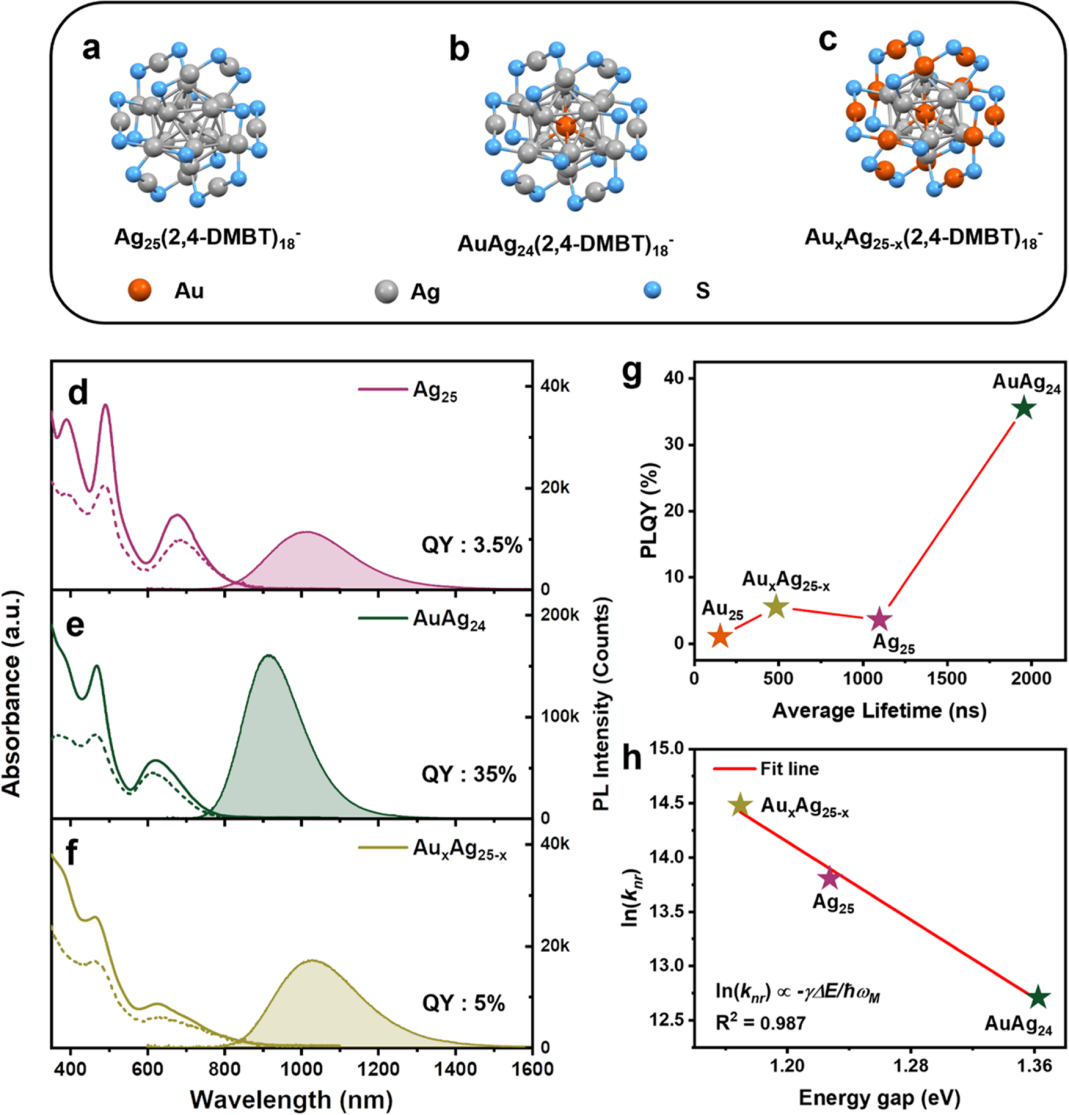
(a) Atomic structure of Ag_25_ (carbon tails and Au(core)–Au(shell) bonds are omitted for clarity). (b) Structure of AuAg_24_. (c) Structure of Au_*x*_Ag_25–*x*_. The structures are redrawn from the previous data.^19,24,53^ Absorption (solid line) and PL (shadowed area) spectra of (d) Ag_25_, (e) AuAg_24_, and (f) Au_*x*_Ag_25–*x*_ in deaerated CDCl_3_ (with N_2_). Dashed lines represent the excitation spectra of PL at (d) 1010 nm, (e) 910 nm, and (f) 1050 nm. For PL measurements: excitation at 500 nm with 0.2 OD, a slit width of 8 nm, and an emission slit of 8 nm. (g) Plot of PLQY versus average lifetimes for the four NCs. (h) Plot of ln(*k*_nr_) versus the photoluminescence gap for the three silver-based NCs. The red line is a linear fit to the energy gap law.

Ag_25_(2,4-DMBT)_18_^–^ and AuAg_24_(2,4-DMBT)_18_^–^ were synthesized by following the protocols developed by the Bakr group.^19,24^ Au_*x*_Ag_25–*x*_(2,4-DMBT)_18_^–^ (*x* = 3–10) was prepared by reacting Ag_25_ with the AuPPh_3_Cl complex.^53^ Besides following the reported procedures, all three silver-based NCs were further extracted by acetonitrile (see the Supporting Information) to improve the purity. Black crystals were obtained by diffusion of hexane into their DCM solutions. As shown in Figure S5, electrospray ionization (ESI) mass spectrometry determined that the number of gold atoms in Au_*x*_Ag_25–*x*_ is between 3 and 10. Based on the previous SCXRD results, the structures of Ag_25_, AuAg_24_, and Au_*x*_Ag_25–*x*_ are shown in Figure 3a–c. Similar to Au_25_, Ag_25_ consists of a Ag_13_ icosahedral kernel and six Ag_2_(2,4-DMBT)_3_ staple motifs.^19^ The doping position of the gold atom in AuAg_24_ is determined to be at the icosahedral center, forming a AuAg_12_ kernel.^24^ In Au_*x*_Ag_25–*x*_, one gold atom occupies the icosahedral center and the rest of the gold atoms substitute silver atoms in the staple motifs. Therefore, Ag_25_ and AuAg_24_ have the same staple motifs but different kernels, while AuAg_24_ and Au_*x*_Ag_25–*x*_ have the same kernel but different staple motifs. Of note, silver NCs are much less stable than gold NCs; thus, we carried out the measurements immediately after we synthesized the samples.

The UV–vis absorption spectra of Ag_25_, AuAg_24_, and Au_*x*_Ag_25–*x*_ NCs in CDCl_3_ (Figure 3d–f, solid lines) are in good agreement with previous reports.^19,24,53^ For comparison with the MAu_24_ series, the steady-state PL spectra (Figure 3d–f, shaded areas) of Ag_25_, AuAg_24_, and Au_*x*_Ag_25–*x*_ NCs were also measured in deaerated CDCl_3_ (with N_2_) using 500 nm excitation. The emission peak of Ag_25_ is found to be at 1000 nm, and it blueshifts to 900 nm for AuAg_24_ and slightly redshifts to 1050 nm for Au_*x*_Ag_25–*x*_. As shown in Figure S6, the peak positions for the silver series in CDCl_3_ are slightly red-shifted compared to their counterparts in DCM. The dashed lines in Figure 3d–f represent the PL excitation spectra of the silver series, and they are consistent with the corresponding UV–vis absorption spectra, suggesting that all the emission is from the first excited state. The absolute PLQY of AuAg_24_ in CDCl_3_ was determined to be 35% using an integrating sphere (Figure S7), and the PLQYs of Ag_25_ and Au_*x*_Ag_25–*x*_ were determined to be 3.5 and 5% by the relative method, respectively.

Time-resolved PL measurements (Figure S8) showed that the decay curves of the silver series can be fitted by monoexponential functions. The PL lifetime of Ag_25_ is determined to be 1100 ns, while the lifetime of AuAg_24_ is prolonged to ∼2000 ns. Surprisingly, Au*_x_*Ag_25–*x*_ has a higher PLQY than Ag_25_, though the lifetime of Au_*x*_Ag_25–*x*_ is shorter (450 ns), indicating that the PL enhancing mechanism is not due to the suppression of nonradiative decay (which would exhibit a prolonged lifetime), but due to the accelerated radiative decay (exhibiting a shorter lifetime or a larger rate). The measured lifetimes are consistent with previous time-correlated single-photon counting (TCSPC) measurements and TAS results.^24,53,54^ The long lifetime suggests the existence of phosphorescence in Ag_25_ and AuAg_24_ NCs, which was previously proved by triplet–triplet annihilation upconversion PL experiments by Niihori et al.,^54^ and the origin of phosphorescence was ascribed to the staple’s triplet state,^54^ but they provided no solid evidence to support that the emission was from the motif states, nor the charge transfer from the core triplet state to the motif triplet state. Previous density functional theory (DFT) calculations proved that the HOMO and LUMO of Ag_25_ were mainly located at the core Ag atoms.^55^ Metal–ligand charge transfer typically requires higher excitation energy and results in a discrepancy between UV–vis absorption spectra and PL excitation spectra.^6,56^ Considering the perfect match of the PL excitation spectra with the UV–vis absorption spectra (Figure 3d–f), we believe that the phosphorescence of the silver series of NCs is from the core state, just like the MAu_24_ series, that is, both pertain to the first excited state.

A plot of PLQY versus the average lifetime for the silver series and Au_25_ is given in Figure 3g. Unlike the gold series, the plot of the silver series does not follow any linear relationship, indicating that the doping of the gold atom(s) into the Ag_25_ template not only affects the nonradiative process but also the radiative rate. Here, since the long lifetime is the main component in the decay curve of the silver series, we could still use  to estimate the radiative rate *k*_r_ and nonradiative rate *k*_nr_. As shown in Figure 3h, similar to the gold series, the silver series also shows a good agreement with the energy gap law, suggesting that the maximum energy of the phonon that assists the nonradiative relaxation is similar among the three silver-based NCs.

### Temperature-Dependent PL Analysis of Ag_25_, AuAg_24_, and Au_*x*_Ag_25–*x*_ (*x* = 3–10) NCs

To further understand the PL mechanism and vibrations of the doped silver NCs, we embedded them into a polystyrene matrix and carried out the temperature-dependent PL measurements from room temperature to 20 K. As shown in Figure S9a–c, the PLQY of all three silver-based NCs is increased in polystyrene films. The emission profiles of Ag_25_ and AuAg_24_ in thin films showed a slight red shift (∼10 nm) compared to their solution counterparts, while the emission peak of Au_*x*_Ag_25–*x*_ shows an 80 nm redshift when embedded in a polystyrene matrix. It suggests that the main contributor (i.e., different numbers of doped gold atoms) to the emission profile of Au_*x*_Ag_25–*x*_ may vary between the solution and thin film. Nevertheless, it is hard to determine the exact species because our synthesis is not able to separate the mixture by the *x* number. The PL lifetimes of the silver series are given in Figure S9d–f, where Ag_25_ possesses a longer lifetime in the thin film, while AuAg_24_ and Au_*x*_Ag_25–*x*_ both have shorter lifetimes in thin films, indicating that both radiative and nonradiative rates are changed.

**Figure 4:**
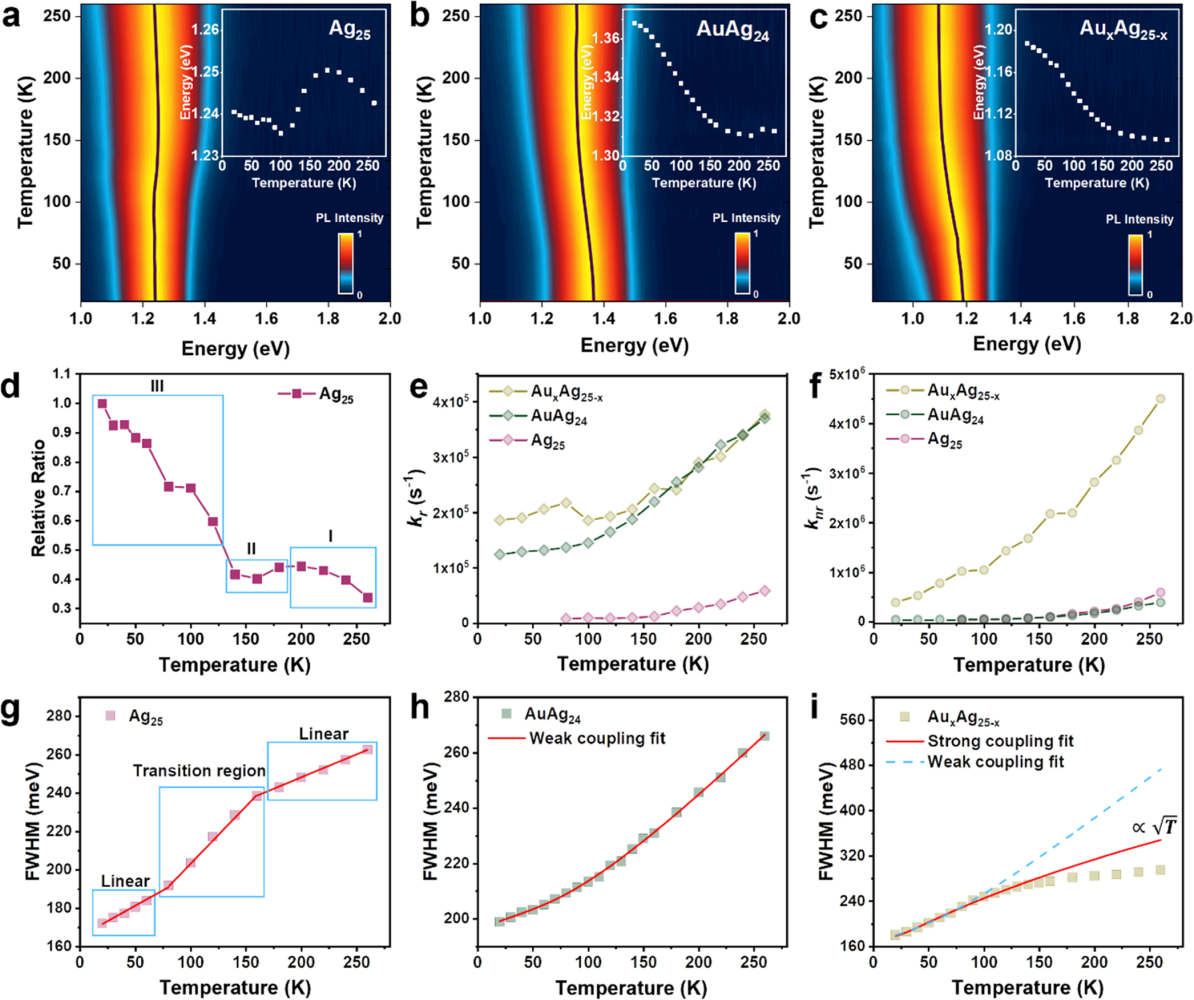
Temperature-dependent PL spectra of (a) Au_*x*_Ag_25–*x*_ (b) AuAg_24_, and (c) Ag_25_ with excitation at 500 nm. (d) Temperature-dependent radiative rate constants of Au_*x*_Ag_25–*x*_, AuAg_24_, and Ag_25_. (d) Normalized integrated PL intensity of Ag_25_ at different temperatures. (e) Temperature-dependent radiative rate of Ag_25_, AuAg_24_, and Au_*x*_Ag_25–*x*_. (f) Temperature-dependent nonradiative rate constants of Ag_25_, AuAg_24_, and Au_*x*_Ag_25–*x*_. The FWHM of the steady-state PL spectra as a function of temperature for (g) Ag_25_, (h) AuAg_24_, and (i) Au_*x*_Ag_25–*x*_. The blue dashed line in panel (i) is the fitting result to eq 4, and the red line is the fitting result to eq 5.

The temperature-dependent PL spectra of the three silver NCs are given in Figure 4a–c, where the black traces and white insets represent the temperature-dependent peak positions. Interestingly, unlike the monotonic trend in the gold series, the temperature-dependent peak position of AuAg_24_ and Ag_25_ show an unusual zig-zag trend as the temperature decreases. Initially, the emission peak undergoes a blue shift, which is followed by a red shift and, eventually, another blue shift. The case of Au_*x*_Ag_25–*x*_ exhibits a monotonic blue shift of the peak position as the temperature decreases; nevertheless, the temperature-dependent peak position presents abnormal flatness between 200 K and room temperature (Figure 4c inset). The plots of temperature-dependent PL intensity for the silver series are given in Figures 4d and S10. The integrated PL intensities of Ag_25_ and AuAg_24_ show a similar three-stage evolution as their temperature-dependent peak positions. In stages I and III (Figure 4d), the PL intensity increases as the temperature decreases, but in stage II (Figure 4d), the PL intensity decreases as the temperature decreases. Meanwhile, a monotonic increase of the integrated PL intensity with decreasing temperature is observed for Au_*x*_Ag_25–*x*_.

Here, we ascribe the abnormal temperature-dependent trends of Ag_25_ and AuAg_24_ to the coexistence of thermally activated delayed fluorescence (TADF) with phosphorescence. This can be rationalized as follows. Taking Ag_25_ as an example, in stage I, both TADF and phosphorescence exist and present a blue shift as the temperature decreases, so the observed peak showed an overall blue shift. The suppression of the nonradiative rate with the temperature decrease results in a net increase of PL intensity in this stage. Then, in stage II, the lower temperature suppresses the reverse intersystem crossing and the TADF starts to vanish, but phosphorescence is still increasing, so the overall peak position showed a red shift, and the integrated PL intensity decreases in this region. In stage III, the TADF is almost completely suppressed, and phosphorescence becomes the major contributor to the emission peak. Therefore, a monotonic blue shift and also a monotonic increase of PL intensity are observed. Compared to Ag_25_, AuAg_24_ shows a less significant decrease of PL intensity in stage II, suggesting a less contribution from TADF in AuAg_24_. The temperature-dependent excitation spectra (Figure S11) of Ag_25_ and AuAg_24_ are consistent with the previously reported temperature-dependent absorption spectra,^57^ suggesting that both TADF and phosphorescence are from the first excited states (S_1_ and T_1_). The case of Au_*x*_Ag_25–*x*_ is much more complicated since it has more than one emitter due to the mixed *x* values. The short lifetime of Au_*x*_Ag_25–*x*_ suggests that its electron dynamic is closer to the MAu_24_ series. Since we do not observe a clear stage II in Au_*x*_Ag_25–*x*_, the TADF component in Au_*x*_Ag_25–*x*_ should be negligible, and phosphorescence is the main contributor, from which we can infer that a full occupation of silver in the six M_2_(SR)_3_ staple motifs is crucial to achieve TADF in the M_25_ NCs. Au_*x*_Ag_25–*x*_ has some gold atoms in the staples, hence, the disappearance of TADF.

The temperature-dependent lifetimes of the silver series are listed in Table S2. Of note, due to the low sensitivity of NIR detectors and the long lifetime of Ag_25_ (>25 μs) at low temperatures, we can only obtain the lifetime of Ag_25_ when the temperature is higher than 80 K. The lifetimes of all three NCs become much prolonged and reach the microsecond scale at low temperatures, suggesting that the emission is from the triplet state. The temperature-dependent radiative rate (Figure 4e) and nonradiative rate (Figure 4f) of the silver series are calculated using the temperature-dependent quantum yields and lifetimes. As shown in Figure 4e, the radiative rates of AuAg_24_ and Au_*x*_Ag_25–*x*_ are close at room temperature, and both are 5 times larger than Ag_25_, suggesting that the central gold atom significantly improves the radiative decay. The radiative rate of Ag_25_ is suppressed to one-fifth at 80 K, which further proves the existence of TADF in Ag_25_.^58^ Meanwhile, the suppression of radiative rates for AuAg_24_ and Au_*x*_Ag_25–*x*_ is much less significant, which is also consistent with our above discussion of the smaller contribution of TADF to these two NCs. The nonradiative rate of Au_*x*_Ag_25–*x*_ is found to be an order of magnitude stronger than the other two silver-based NCs, suggesting that the Au_2_(SR)_3_ staple motif is the main origin of nonradiative relaxation and that partial substitution by Ag_2_(SR)_3_ is not able to alleviate the fast nonradiative relaxation. As the temperature decreases, the nonradiative rate of the silver-based NCs is suppressed significantly. The nonradiative rates of Ag_25_ and AuAg_24_ are very close at all temperatures, indicating that the central gold doping has a trivial effect on the vibration.

To better understand the vibrational properties of the three silver NCs, we extracted the temperature-dependent linewidths of the silver series from their corresponding emission spectra, and the plots are given in Figure 4g–i. Due to the large component of TADF at room temperature for Ag_25_, its temperature-dependent linewidth (Figure 4g) also presents a three-stage evolution that shares similar transition temperatures as the temperature-dependent peak position and intensity. Interestingly, when the temperature is above 160 K (mainly TADF), we observe a linear relationship between the temperature and PL linewidth, suggesting a weak electron–phonon interaction in Ag_25_. Meanwhile, a similar linear relationship is observed when the temperature is lower than 80 K (mainly phosphorescence), suggesting that the acoustic phonon is the main contributor to the PL broadening at low temperatures.^43^ However, the overall zig-zag trend of Ag_25_ prohibits us from doing further quantitative analyses. Fortunately, in the case of AuAg_24_, less TADF and higher transition temperature lead to less interference in the analysis of temperature-dependent PL linewidths. As shown in Figure 4h, the temperature-dependent linewidth of AuAg_24_ can be well fitted by the weak e–ph coupling model given in eq 4 (the last term is neglected since no impurity exists), and Table 2 gives the extracted parameters. The energy of the optical phonon is determined to be 28 meV, which matches well with the vibration energy of Ag_2_(SR)_3_ staple motifs determined by Raman spectroscopy.^59^ In Figure 4i, the temperature-dependent linewidth of Au*_x_*Ag_25–*x*_ is similar to the MAu_24_ series, which shows a nonlinear relationship at high temperatures. However, the multiple emitters in Au_*x*_Ag_25–*x*_ due to different *x* make it not possible to fit the temperature-dependent linewidth by the strong coupling model. The clear nonlinear relationship in the high-temperature region is sufficient to prove the strong electron–phonon interaction in Au_*x*_Ag_25–*x*_;^45^ hence, its less PLQY, albeit the radiative rate is equally accelerated by central gold doping as in AuAg_24_, that is, the strong e–ph coupling from the gold-containing staples significantly counteracts the role of *k*_r_ enhancement and thus reduces the PLQY of Au_*x*_Ag_25–*x*_ as in the gold series.

**Table 2:** Extracted Parameters for AuAg_24_ (Fitted by eq 4 without the Last Term)

	Γ_0_, meV	γ_ac_, meV·K^–1^	γ_LO_, meV	*E*_LO_, meV
AuAg_24_	196	0.138	86	28

**Scheme 2:**
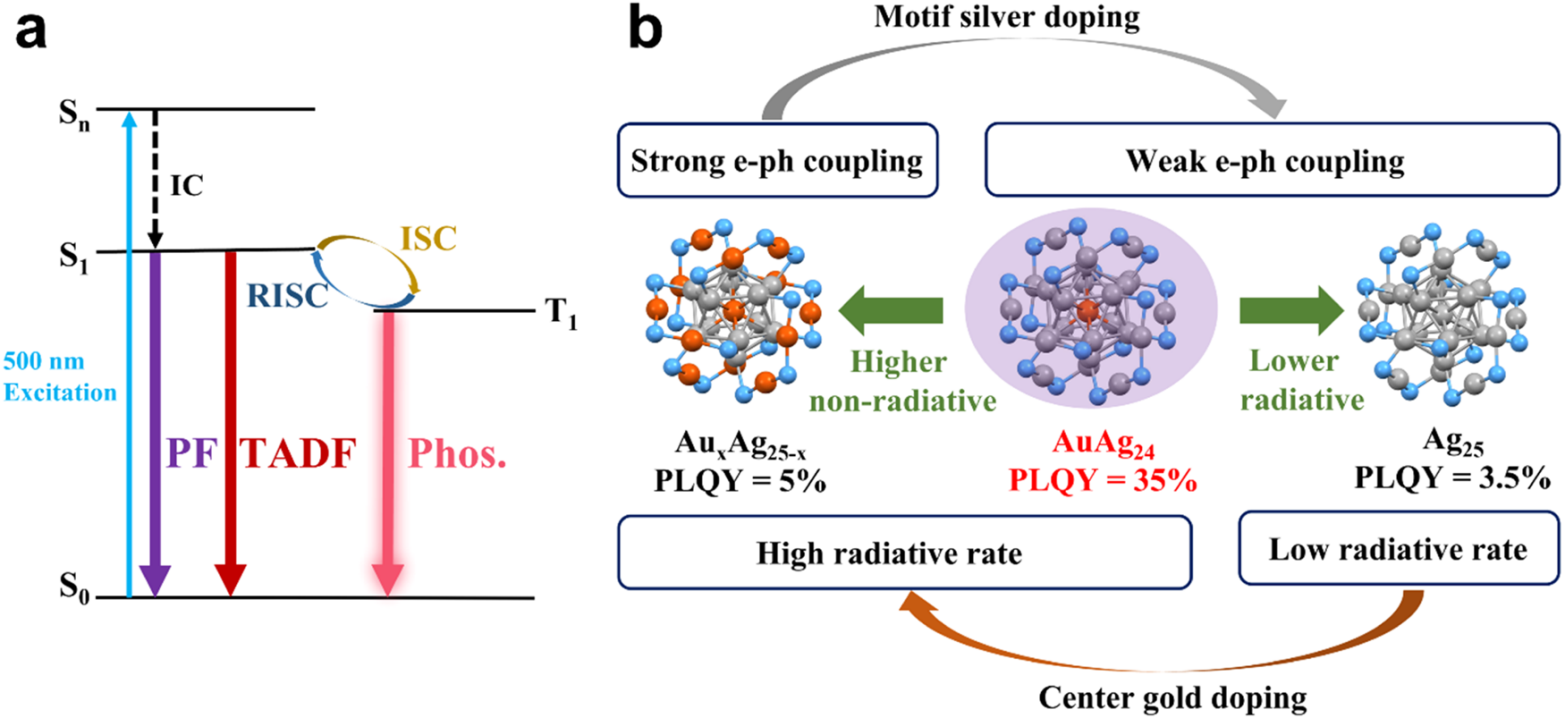
(a) Emission Mechanism of Ag_25_ and AuAu_24_. (b) Summary of Doping Effects in the Ag_25_-Based Series of NCs

By combining the discussions above, the emission mechanism of Ag_25_ and AuAg_24_ is summarized in Scheme 2a, whereas the case of Au_*x*_Ag_25–*x*_ is closer to Scheme 1 because it exhibits predominant phosphorescence. Since the excitation spectra of the silver series match well with their corresponding UV–vis absorption spectra, Kasha’s rule is valid. The different electron dynamics between Au_*x*_Ag_25–*x*_ and Ag_25_/AuAg_24_ indicate that a full occupation of M_2_(SR)_3_ staple motifs by Ag atoms is critical for observing TADF. Of note, although the percentage of TADF contributing to the emission intensity of Ag_25_ is greater than that in AuAg_24_, it does not necessarily prove that the reverse intersystem crossing is more efficient in Ag_25_ because the overall PLQY of AuAg_24_ is 10 times higher than Ag_25_. The reverse intersystem crossing should also exist in Au_*x*_Ag_25–*x*_ or even the gold series, but the strong e–ph interaction in the latter series leads to an efficient nonradiative relaxation that completely suppresses the radiative recombination. In other words, the change from strong e–ph coupling in the gold series to weak e–ph coupling in the silver series (except Au*_x_*Ag_25–*x*_) should be the real reason that TADF is observed in Ag_25_ and AuAg_24_.

The doping effects of gold atoms in the Ag_25_ template are summarized in Scheme 2b. When gold atoms replace silver atoms in the staple motifs, it introduces strong electron–phonon coupling and results in fast nonradiative relaxation. The correlation between Au_2_(SR)_3_ and strong e–ph interactions is consistent with the afore-discussed results of the MAu_24_ series. The strong coupling may originate from the heavy mass and rich orbitals of gold atoms. Interestingly, the central doping by the gold atom significantly increases the radiative decay in AuAg_24_ and Au_*x*_Ag_25–*x*_. Such a phenomenon cannot be explained by previous DFT simulations because no triplet state was taken into consideration. Therefore, further theoretical studies are needed in order to better understand the excited states of the silver series. In the case of AuAg_24_, the six Ag_2_(SR)_3_ staple motifs help the decoupling between the electron and phonons (i.e., suppressing the nonradiative decay), and the central gold atom boosts the radiative decay. These two factors make AuAg_24_ one of the most luminescent nanoclusters (QY = 35% at r.t.).

Finally, we compile the PL data of the gold series in Table 3 and the silver series in Table 4 for ease of comparison.

**Table 3:** PL Parameters of the Three Au-Based NCs at Different Temperatures

	Au_25_	CdAu_24_	HgAu_24_
solution at 298 K			
peak position (eV)	1.14	1.19	1.06
lifetime (ns)	155	257	53
PLQY (%)	1	1.7	0.3
film at 290 K			
peak position (eV)	1.13	1.16	1.06
lifetime (ns)	202	275	99
PLQY (%)	1.8	7	2.2
film at 160 K			
peak position (eV)	1.15	1.18	1.07
lifetime (ns)	421	633	293
PLQY (%)	2.4	9.5	6.4
film at 20 K			
peak position (eV)	1.17	1.21	1.09
lifetime (ns)	921	2817	720
PLQY (%)	6	25	14

**Table 4:** PL Parameters of the Three Ag-Based NCs at Different Temperatures

	Ag_25_	AuAg_24_	Au_*x*_Ag_25–*x*_
solution at 298 K			
peak position (eV)	1.23	1.36	1.18
lifetime (ns)	1100	1957	450
PLQY (%)	3.5	35	5
film at 280 K			
peak position (eV)	1.24	1.31	1.09
lifetime (ns)	1296	1200	229
PLQY (%)	9	43	7
film at 160 K			
peak position (eV)	1.25	1.32	1.11
lifetime (ns)	9794	3192	413
PLQY (%)	10	70	10
film at 20 K			
peak position (eV)	1.24	1.37	1.19
lifetime (ns)	>25 000	6132	1414
PLQY (%)	30	76	26

## Conclusions

In summary, we have systematically studied the PL properties for a series of superatomic closed shell (1S^2^|1P^6^ electron configuration) M_25_ NCs. The ∼1100 nm NIR emission of the three Au_25_-based NCs is determined as phosphorescence. Heteroatom doping by the Cd or Hg atom is found to mainly affect the nonradiative process (i.e., suppressing the electron–acoustic phonon interaction) rather than affecting the radiative relaxation. An unusually strong electron–phonon interaction is found to exist in all three Au_25_-based NCs, and the optical phonon from the Au_2_(SR)_3_ vibration is determined as the main contributor. In the second system (the three Ag_25_-based NCs), the electron–phonon interaction is found to be weakened from heavy gold doped Au_*x*_Ag_25–*x*_ to the original Ag_25_ NC, which further supports that the Au_2_(SR)_3_ staple motif is the main source for strong electron–phonon interactions. Due to the much weaker electron–phonon interaction in Ag_25_ and AuAg_24_, the carrier lifetime is much prolonged, and we observe the existence of TADF in the emission profile of these two NCs. Interestingly, the center doping of gold atoms into Ag_25_ significantly improves the radiative recombination. The efficient radiative relaxation from the central gold atom and the suppression of nonradiative relaxation by six Ag_2_(SR)_3_ staple motifs make AuAg_24_ one of the most luminescent NCs. These insights can be extended to other icosahedral NCs, such as Au_38_ and bi-icosahedral Au_25_, and will facilitate the development of applications of luminescent metal nanoclusters.

## Supporting Information Available

Details of the synthesis, spectroscopy, and data analysis, Figures S1–S11, and Tables S1 and S2 (PDF)

## References

[ref1] Du B.; Jiang X.; Das A.; Zhou Q.; Yu M.; Jin R.; Zheng J. Glomerular Barrier Behaves as an Atomically Precise Bandpass Filter in a Sub-Nanometre Regime. Nat. Nanotechnol. 2017, 12, 1096–1102. 10.1038/nnano.2017.170. 28892099 PMC5679252

[ref2] Huang H.-Y.; Cai K.-B.; Chen P.-W.; Lin C.-A. J.; Chang S.-H.; Yuan C.-T. Engineering Ligand–Metal Charge Transfer States in Cross-Linked Gold Nanoclusters for Greener Luminescent Solar Concentrators with Solid-State Quantum Yields Exceeding 50% and Low Reabsorption Losses. J. Phys. Chem. C 2018, 122, 20019–20026. 10.1021/acs.jpcc.8b06212.

[ref3] Zhu M.; Yao Q.; Liu Z.; Liu J.; Liu M.; Long M.; Xie J. Aggregation-Induced Emission of Gold Nanoclusters by Ionic Liquids for White Light-Emitting Diode and Multiple-Ion Probe Applications. J. Phys. Chem. Lett. 2022, 13, 7722–7730. 10.1021/acs.jpclett.2c02042. 35969058

[ref4] Liu Z.; Li Y.; Kahng E.; Xue S.; Du X.; Li S.; Jin R. Tailoring the Electron–Phonon Interaction in Au_25_(SR)_18_ Nanoclusters via Ligand Engineering and Insight into Luminescence. ACS Nano 2022, 16, 18448–18458. 10.1021/acsnano.2c06586. 36252530

[ref5] Zhong Y.; Zhang J.; Li T.; Xu W.; Yao Q.; Lu M.; Bai X.; Wu Z.; Xie J.; Zhang Y. Suppression of Kernel Vibrations by Layer-by-Layer Ligand Engineering Boosts Photoluminescence Efficiency of Gold Nanoclusters. Nat. Commun. 2023, 14, 658 10.1038/s41467-023-36387-2. 36746958 PMC9902523

[ref6] Zhou M.; Song Y. Origins of Visible and Near-Infrared Emissions in [Au_25_(SR)_18_]^−^ Nanoclusters. J. Phys. Chem. Lett. 2021, 12, 1514–1519. 10.1021/acs.jpclett.1c00120. 33534598

[ref7] Wu Z.; Jin R. On the Ligand’s Role in the Fluorescence of Gold Nanoclusters. Nano Lett. 2010, 10, 2568–2573. 10.1021/nl101225f. 20550101

[ref8] Gan Z.; Lin Y.; Luo L.; Han G.; Liu W.; Liu Z.; Yao C.; Weng L.; Liao L.; Chen J.; et al. Fluorescent Gold Nanoclusters with Interlocked Staples and a Fully Thiolate-Bound Kernel. Angew. Chem., Int. Ed. 2016, 55, 11567–11571. 10.1002/anie.201606661. 27529838

[ref9] Chakraborty S.; Bain D.; Maity S.; Kolay S.; Patra A. Controlling Aggregation-Induced Emission in Bimetallic Gold–Copper Nanoclusters via Surface Motif Engineering. J. Phys. Chem. C 2022, 126, 2896–2904. 10.1021/acs.jpcc.1c10237.

[ref10] Yao C.; Xu C. Q.; Park I. H.; Zhao M.; Zhu Z.; Li J.; Hai X.; Fang H.; Zhang Y.; Macam G.; et al. Giant Emission Enhancement of Solid-State Gold Nanoclusters by Surface Engineering. Angew. Chem., Int. Ed. 2020, 59, 8270–8276. 10.1002/anie.202001034. 32003098

[ref11] Zhang S.-S.; Havenridge S.; Zhang C.; Wang Z.; Feng L.; Gao Z.-Y.; Aikens C. M.; Tung C.-H.; Sun D. Sulfide Boosting Near-Unity Photoluminescence Quantum Yield of Silver Nanocluster. J. Am. Chem. Soc. 2022, 144, 18305–18314. 10.1021/jacs.2c06093. 36169057

[ref12] Zhu C.; Xin J.; Li J.; Li H.; Kang X.; Pei Y.; Zhu M. Fluorescence or Phosphorescence? The Metallic Composition of the Nanocluster Kernel Does Matter. Angew. Chem., Int. Ed. 2022, 61, e202205947 10.1002/anie.202205947. 35596616

[ref13] Takano S.; Hirai H.; Nakashima T.; Iwasa T.; Taketsugu T.; Tsukuda T. Photoluminescence of Doped superatoms M@Au_12_ (M = Ru, Rh, Ir) Homoleptically Capped by (Ph_2_)PCH_2_P(Ph_2_): Efficient Room-Temperature Phosphorescence from Ru@Au_12_. J. Am. Chem. Soc. 2021, 143, 10560–10564. 10.1021/jacs.1c05019. 34232036

[ref14] Hirai H.; Takano S.; Nakashima T.; Iwasa T.; Taketsugu T.; Tsukuda T. Doping-Mediated Energy-Level Engineering of M@Au_12_ Superatoms (M = Pd, Pt, Rh, Ir) for Efficient Photoluminescence and Photocatalysis. Angew. Chem., Int. Ed. 2022, 61, e202207290 10.1002/ange.202207290. 35608869

[ref15] Mitsui M.; Arima D.; Kobayashi Y.; Lee E.; Niihori Y. On the Origin of Photoluminescence Enhancement in Biicosahedral Ag_x_Au_25–x_ Nanoclusters (x = 0–13) and Their Application to Triplet–Triplet Annihilation Photon Upconversion. Adv. Opt. Mater. 2022, 10, 2200864 10.1002/adom.202200864.

[ref16] Aikens C. M. Electronic and Geometric Structure, Optical Properties, and Excited State Behavior in Atomically Precise Thiolate-Stabilized Noble Metal Nanoclusters. Acc. Chem. Res. 2018, 51, 3065–3073. 10.1021/acs.accounts.8b00364. 30444598

[ref17] Zhu M.; Aikens C. M.; Hollander F. J.; Schatz G. C.; Jin R. Correlating the Crystal Structure of a Thiol-Protected Au_25_ Cluster and Otical Poperties. J. Am. Chem. Soc. 2008, 130, 5883–5885. 10.1021/ja801173r. 18407639

[ref18] Heaven M. W.; Dass A.; White P. S.; Holt K. M.; Murray R. W. Crystal Structure of the Gold Nanoparticle [N(C_8_H_17_)_4_][Au_25_(SCH_2_CH_2_Ph)_18_]. J. Am. Chem. Soc. 2008, 130, 3754–3755. 10.1021/ja800561b. 18321116

[ref19] Joshi C. P.; Bootharaju M. S.; Alhilaly M. J.; Bakr O. M. [Ag_25_(SR)_18_]^−^: The “Golden” Silver Nanoparticle. J. Am. Chem. Soc. 2015, 137, 11578–11581. 10.1021/jacs.5b07088. 26322865

[ref20] Qian H.; Jiang D.-e.; Li G.; Gayathri C.; Das A.; Gil R. R.; Jin R. Monoplatinum Doping of Gold Nanoclusters and Catalytic Application. J. Am. Chem. Soc. 2012, 134, 16159–16162. 10.1021/ja307657a. 22992034

[ref21] Suyama M.; Takano S.; Nakamura T.; Tsukuda T. Stoichiometric Formation of Open-Shell [PtAu_24_(SC_2_H_4_Ph)_18_]^−^ via Spontaneous Electron Proportionation between [PtAu_24_(SC_2_H_4_Ph)_18_]^2–^ and [PtAu_24_(SC_2_H_4_Ph)_18_]^0^. J. Am. Chem. Soc. 2019, 141, 14048–14051. 10.1021/jacs.9b06254. 31403779

[ref22] Suyama M.; Takano S.; Tsukuda T. Synergistic Effects of Pt and Cd Codoping to Icosahedral Au_13_ Superatoms. J. Phys. Chem. C 2020, 124, 23923–23929. 10.1021/acs.jpcc.0c06765.

[ref23] Li Y.; Biswas S.; Luo T.-Y.; Juarez-Mosqueda R.; Taylor M. G.; Mpourmpakis G.; Rosi N. L.; Hendrich M. P.; Jin R. Doping Effect on the Magnetism of Thiolate-Capped 25-Atom Alloy Nanoclusters. Chem. Mater. 2020, 32, 9238–9244. 10.1021/acs.chemmater.0c02984.

[ref24] Bootharaju M. S.; Joshi C. P.; Parida M. R.; Mohammed O. F.; Bakr O. M. Templated Atom-Precise Galvanic Synthesis and Structure Elucidation of a [Ag_24_Au(SR)_18_]^−^ Nanocluster. Angew. Chem. 2016, 128, 934–938. 10.1002/ange.201509381. 26611172

[ref25] Yan J.; Su H.; Yang H.; Malola S.; Lin S.; Häkkinen H.; Zheng N. Total Structure and Electronic Structure Analysis of Doped Thiolated Silver [MAg_24_(SR)_18_]^2–^(M = Pd, Pt) Clusters. J. Am. Chem. Soc. 2015, 137, 11880–11883. 10.1021/jacs.5b07186. 26351859

[ref26] Wu Z.; Suhan J.; Jin R. One-Pot Synthesis of Atomically Monodisperse, Thiol-Functionalized Au_25_ Nanoclusters. J. Mater. Chem. 2009, 19, 622–626. 10.1039/B815983A.

[ref27] Fei W.; Antonello S.; Dainese T.; Dolmella A.; Lahtinen M.; Rissanen K.; Venzo A.; Maran F. Metal Doping of Au_25_(SR)_18_^–^ Clusters: Insights and Hindsights. J. Am. Chem. Soc. 2019, 141, 16033–16045. 10.1021/jacs.9b08228. 31532209

[ref28] Wang S.; Song Y.; Jin S.; Liu X.; Zhang J.; Pei Y.; Meng X.; Chen M.; Li P.; Zhu M. Metal Exchange Method Using Au_25_ Nanoclusters as Templates for Alloy Nanoclusters with Atomic Precision. J. Am. Chem. Soc. 2015, 137, 4018–4021. 10.1021/ja511635g. 25799517

[ref29] Liao L.; Zhou S.; Dai Y.; Liu L.; Yao C.; Fu C.; Yang J.; Wu Z. Mono-mercury Doping of Au_25_ and the HOMO/LUMO Energies Evaluation Employing Differential Pulse Voltammetry. J. Am. Chem. Soc. 2015, 137, 9511–9514. 10.1021/jacs.5b03483. 26196263

[ref30] Yao C.; Lin Y.-j.; Yuan J.; Liao L.; Zhu M.; Weng L.-h.; Yang J.; Wu Z. Mono-cadmium vs Mono-mercury Doping of Au_25_ Nanoclusters. J. Am. Chem. Soc. 2015, 137, 15350–15353. 10.1021/jacs.5b09627. 26595532

[ref31] Peng J.; Huang B.; Wang P.; Pei Y. On the Mechanism of Anti-galvanic Metal Displacement Reaction between [Au_25_(SR)_18_]^−^ and Metal-Thiolate Complex. J. Phys. Chem. A 2022, 126, 8910–8917. 10.1021/acs.jpca.2c04948. 36413485

[ref32] Zhou M.; Yao C.; Sfeir M. Y.; Higaki T.; Wu Z.; Jin R. Excited-State Behaviors of M_1_Au_24_(SR)_18_ Nanoclusters: The Number of Valence Electrons Matters. J. Phys. Chem. C 2018, 122, 13435–13442. 10.1021/acs.jpcc.7b11057.

[ref33] Kawasaki H.; Kumar S.; Li G.; Zeng C.; Kauffman D. R.; Yoshimoto J.; Iwasaki Y.; Jin R. Generation of Singlet Oxygen by Photoexcited Au_25_(SR)_18_ Clusters. Chem. Mater. 2014, 26, 2777–2788. 10.1021/cm500260z.

[ref34] Agrachev M.; Fei W.; Antonello S.; Bonacchi S.; Dainese T.; Zoleo A.; Ruzzi M.; Maran F. Understanding and Controlling the Efficiency of Au_24_M(SR)_18_ Nanoclusters as Singlet-Oxygen Photosensitizers. Chem. Sci. 2020, 11, 3427–3440. 10.1039/D0SC00520G. 34777743 PMC8524663

[ref35] Shafikov M. Z.; Daniels R.; Kozhevnikov V. N. Unusually Fast Phosphorescence from Ir (III) Complexes via Dinuclear Molecular Design. J. Phys. Chem. Lett. 2019, 10, 7015–7024. 10.1021/acs.jpclett.9b03002. 31638816

[ref36] Qian H.; Sfeir M. Y.; Jin R. Ultrafast Relaxation Dynamics of [Au_25_(SR)_18_]^*q*^ Nanoclusters: Effects of Charge State. J. Phys. Chem. C 2010, 114, 19935–19940. 10.1021/jp107915x.

[ref37] Wei Y.-C.; Wang S. F.; Hu Y.; Liao L.-S.; Chen D.-G.; Chang K.-H.; Wang C.-W.; Liu S.-H.; Chan W.-H.; Liao J.-L.; et al. Overcoming the Energy Gap Law in Near-Infrared OLEDs by Exciton–Vibration Decoupling. Nat. Photonics 2020, 14, 570–577. 10.1038/s41566-020-0653-6.

[ref38] Kwak K.; Thanthirige V. D.; Pyo K.; Lee D.; Ramakrishna G. Energy Gap Law for Exciton Dynamics in Gold Cluster Molecules. J. Phys. Chem. Lett. 2017, 8, 4898–4905. 10.1021/acs.jpclett.7b01892. 28933858

[ref39] Liu Z.; Li Y.; Shin W.; Jin R. Observation of Core Phonon in Electron–Phonon Coupling in Au_25_ Nanoclusters. J. Phys. Chem. Lett. 2021, 12, 1690–1695. 10.1021/acs.jpclett.1c00050. 33560861

[ref40] Aizawa N.; Pu Y.-J.; Harabuchi Y.; Nihonyanagi A.; Ibuka R.; Inuzuka H.; Dhara B.; Koyama Y.; Nakayama K.-i.; Maeda S.; et al. Delayed Fluorescence from Inverted Singlet and Triplet Excited States. Nature 2022, 609, 502–506. 10.1038/s41586-022-05132-y. 36104553 PMC9477729

[ref41] Rudin S.; Reinecke T.; Segall B. Temperature-Dependent Exciton Linewidths in Semiconductors. Phys. Rev. B 1990, 42, 11218 10.1103/PhysRevB.42.11218. 9995407

[ref42] Alivisatos A. P.; Harris A.; Levinos N.; Steigerwald M.; Brus L. Electronic States of Semiconductor Clusters: Homogeneous and Inhomogeneous Broadening of the Optical Spectrum. J. Chem. Phys. 1988, 89, 4001–4011. 10.1063/1.454833.

[ref43] Wright A. D.; Verdi C.; Milot R. L.; Eperon G. E.; Pérez-Osorio M. A.; Snaith H. J.; Giustino F.; Johnston M. B.; Herz L. M. Electron–Phonon Coupling in Hybrid Lead Halide Perovskites. Nat. Commun. 2016, 7, 11755 10.1038/ncomms11755. PMC4894981 27225329

[ref44] Lee J.; Koteles E. S.; Vassell M. Luminescence Linewidths of Excitons in GaAs Quantum Wells below 150 K. Phys. Rev. B 1986, 33, 5512 10.1103/PhysRevB.33.5512. 9939057

[ref45] Vuong T. Q. P.; Cassabois G.; Valvin P.; Liu S.; Edgar J.; Gil B. Exciton-Phonon Interaction in the Strong-Coupling Regime in Hexagonal Boron Nitride. Phys. Rev. B 2017, 95, 201202 10.1103/PhysRevB.95.201202.

[ref46] Wu B.; Ning W.; Xu Q.; Manjappa M.; Feng M.; Ye S.; Fu J.; Lie S.; Yin T.; Wang F.; et al. Strong Self-Trapping by Deformation Potential Limits Photovoltaic Performance in Bismuth Double Perovskite. Sci. Adv. 2021, 7, eabd3160 10.1126/sciadv.abd3160. 33597239 PMC7888938

[ref47] Toyozawa Y. Theory of Line-Shapes of the Exciton Absorption Bands. Prog. Theor. Phys. 1958, 20, 53–81. 10.1143/PTP.20.53.

[ref48] Maioli P.; Stoll T.; Sauceda H. E.; Valencia I.; Demessence A.; Bertorelle F.; Crut A.; Vallee F.; Garzon I. L.; Cerullo G.; Del Fatti N. Mechanical Vibrations of Atomically Defined Metal Clusters: From Nano- to Molecular-Size Oscillators. Nano Lett. 2018, 18, 6842–6849. 10.1021/acs.nanolett.8b02717. 30247927

[ref49] Bürgi T. Properties of the Gold–Sulphur Interface: from Self-Assembled Monolayers to Clusters. Nanoscale 2015, 7, 15553–15567. 10.1039/C5NR03497C. 26360607

[ref50] Yu X.; Sun Y.; Xu W.-w.; Fan J.; Gao J.; Jiang X.; Su Y.; Zhao J. Tuning Photoelectron Dynamic Behavior of Thiolate-Protected MAu_24_ Nanoclusters via Heteroatom Substitution. Nanoscale Horiz. 2022, 7, 1192–1200. 10.1039/D2NH00281G. 36039937

[ref51] Krishnadas K. R.; Baghdasaryan A.; Kazan R.; Banach E.; Teyssier J.; Nicu V. P.; Buergi T. Raman Spectroscopic Fingerprints of Atomically Precise Ligand Protected Noble Metal Clusters: Au_38_(PET)_24_ and Au_38–x_Ag_x_(PET)_24_. Small 2021, 17, 2101855 10.1002/smll.202101855. 34405952

[ref52] Krishnadas K. R.; Ghosh D.; Ghosh A.; Natarajan G.; Pradeep T. Structure–Reactivity Correlations in Metal Atom Substitutions of Monolayer-Protected Noble Metal Alloy Clusters. J. Phys. Chem. C 2017, 121, 23224–23232. 10.1021/acs.jpcc.7b07605.

[ref53] Pniakowska A.; Kumaranchira Ramankutty K.; Obstarczyk P.; Perić Bakulić M.; Sanader Maršić Ž.; Bonačić-Koutecký V.; Bürgi T.; Olesiak-Banska J. Gold-Doping Effect on Two-Photon Absorption and Luminescence of Atomically Precise Silver Ligated Nanoclusters. Angew. Chem., Int. Ed. 2022, 61, e202209645 10.1002/anie.202209645. 36005739

[ref54] Niihori Y.; Wada Y.; Mitsui M. Single Platinum Atom Doping to Silver Clusters Enables Near-Infrared-to-Blue Photon Upconversion. Angew. Chem., Int. Ed. 2021, 60, 2822–2827. 10.1002/anie.202013725. 33295118

[ref55] Weerawardene K. L. D. M.; Aikens C. M. Origin of Photoluminescence of Ag_25_(SR)_18_^–^ Nanoparticles: Ligand and Doping Effect. J. Phys. Chem. C 2018, 122, 2440–2447. 10.1021/acs.jpcc.7b11706.

[ref56] Si W.-D.; Zhang C.; Zhou M.; Tian W.-D.; Wang Z.; Hu Q.; Song K.-P.; Feng L.; Huang X.-Q.; Gao Z.-Y.; et al. Two Triplet Emitting States in One Emitter: Near-Infrared Dual-Phosphorescent Au_20_ Nanocluster. Sci. Adv. 2023, 9, eadg3587 10.1126/sciadv.adg3587. 36989358 PMC10058230

[ref57] Yuan Q.; Kang X.; Hu D.; Qin C.; Wang S.; Zhu M. Metal Synergistic Effect on Cluster Optical Properties: Based on Ag_25_ Series Nanoclusters. Dalton Trans. 2019, 48, 13190–13196. 10.1039/C9DT02493J. 31414093

[ref58] Hofbeck T.; Monkowius U.; Yersin H. Highly Efficient Luminescence of Cu (I) Compounds: Thermally Activated Delayed Fuorescence Combined with Short-Lived Phosphorescence. J. Am. Chem. Soc. 2015, 137, 399–404. 10.1021/ja5109672. 25486064

[ref59] Ramankutty K. K.; Yang H.; Baghdasaryan A.; Teyssier J.; Nicu V. P.; Buergi T. Molecule-Like and Lattice Vibrations in Metal Clusters. Phys. Chem. Chem. Phys. 2022, 24, 13848–13859. 10.1039/D1CP04708F. 35616625 PMC9176185

