# Supplementary material for: Elucidating the Near-Infrared Photoluminescence Mechanism of Homometal and Doped M25(SR)18 Nanoclusters
Source: J Am Chem Soc. 2023 Aug 29;145(36):19969–81. doi: 10.1021/jacs.3c06543 (PMC10510323; doi:10.1021/jacs.3c06543)
Supplement: Supplementary file 1 — ja3c06543_si_001.pdf [file ja3c06543_si_001.pdf]

(Supporting Information)

Elucidating the Near-Infrared Photoluminescence Mechanism of Homometal and Doped $M_{25}(SR)_{18}$ Nanoclusters

Zhongyu Liu, Meng Zhou, Lianshun Luo, Yitong Wang, Ellen Kahng, and Rongchao Jin*

Department of Chemistry, Carnegie Mellon University, Pittsburgh Pennsylvania 15213, USA

*Correspondence author, Email: rongchao@andrew.cmu.edu

Experimental/Methods

Chemicals. Tetrachloroauric(III) acid (HAuCl4·3H2O, >99.99% metal basis, Aldrich), silver nitride (99%, Aldrich), tetraoctylammonium bromide (TOAB, ≥98%, Aldrich), 2-phenylethanethiol (PET, 98%, Aldrich), 2,4-dimethylbenzenethiol (2,4-DMBT, 98%, TCI), tetraphenylphosphonium bromide (98%, TCI), polystyrene (PS, Aldrich). Solvents: 2-Methyltetrahydrofuran, deuterated chloroform, methanol, ethanol, dichloromethane (DCM), chloroform, acetone, hexane, and acetonitrile (HPLC grade for all solvents). All chemicals were used without further purification. Deionized water was prepared with Barnstead NANOpure Diamond (18.2MΩ cm). Thin-layer chromatography (TLC) plates (iChromatography, silica gel, 250 μm).

Synthesis of $Au_{25}(PET)_{18}^-$: $Au_{25}(PET)_{18}^-$ was synthesized following a previously reported one-pot size-focusing protocol.[s1] First, $HAuCl_4·3H_2O$ (0.2 mmol, 80mg) and TOAB (0.233 mmol, 127mg) were dissolved in 15 mL tetrahydrofuran at room temperature and stirred for 15 min. The solution gradually turned from light yellow to reddish-orange. After that, 140 μL 2-phenylethanethiol was added and stirred vigorously until the solution turned colorless. Then, a freshly made $NaBH_4$ solution (2 mmol, 77 mg dissolved in 5mL of cold deionized water) was rapidly added to the mixture. The solution immediately turned black, and the reaction was allowed to proceed overnight. The crude product was washed with methanol four times to remove the excess thiol and inorganic impurities. $Au_{25}(PET)_{18}^-$ nanoclusters were extracted from the residue with acetonitrile.

Synthesis of $CdAu_{24}(PET)_{18}$: $CdAu_{24}(PET)_{18}$ was synthesized using a reported method with small modification.[2-3] $Cd(NO_3)_2$ (308.47 mg, 1 mmol) was first dissolved in 5 mL methanol. Then, phenylethanethiol (0.67 mL, 5 mmol) and triethylamine (2 mL, 15 mmol) in 5 mL methanol was added. A white floc was seen at first but re-dissolved immediately. After an hour, the solvent was removed by rotary evaporation and the white precipitate was collected. The crude products were then washed with H_2O (5 × 10 mL) and methanol (5 × 10 mL) to obtain $Cd(PET)_2$ as a white powder. To obtain $CdAu_{24}(PET)_{18}$, 10 mg $Au_{25}(SC_2Ph)_{18}^-$ was dissolved in 5 mL of toluene at 55 °C and 0.4 mg aliquots of $Cd(PET)_2$ was added (as a powder) to the solution every hour, for a total of 10 times. The reaction was allowed to proceed for at least 50 hours, and a black crude product was obtained after removing the solvent. After washing the sample with methanol three times, pure $CdAu_{24}(PET)_{18}$ was separated by TLC. With different reaction times, the best developing solvent for TLC separation may vary from 1:3 (v/v) DCM:*n*-hexane to 1:6 (v/v) DCM:*n*-hexane. Pure $CdAu_{24}(PET)_{18}$ is an olive green band on the TLC plate.

Synthesis of $HgAu_{24}(PET)_{18}$: $HgAu_{24}(PET)_{18}$ was synthesized by a reported method with small modifications.[s2-3] First, $Hg(PET)_2$ was synthesized using the same protocol as we described in the synthesis of $Cd(PET)_2$ but adopting $Hg(NO_3)_2$ (324.6 mg, 1 mmol) as the precursor. $HgAu_{24}(PET)_{18}$ was synthesized

by mixing 10 mg $Au_{25}(PET)_{18}^{-}$ (in 5 mL toluene) with 2 mg $Hg(PET)_2$ under vigorous stirring. The reaction was allowed to proceed for 20 min. The organic layer was separated from excess $Hg(PET)_2$ and the solution was evaporated to dryness. After washing with methanol three times, pure $HgAu_{24}(PET)_{18}$ was separated by TLC. In our case, the best developing solvent in TLC separation is 1:1 (v/v) DCM:n-hexane. A second round TLC separtaion may be needed to obtain high purity products. Pure $HgAu_{24}(PET)_{18}$ is a dark yellow band on the TLC plate.

Synthesis of $Ag_{25}(2,4\text{-}DMBT)_{18}^{-}$: $Ag_{25}(2,4\text{-}DMBT)_{18}^{-}$ was synthesized following a previously reported protocol with small modifications.[s4] First, 38 mg of $AgNO_3$ was dissolved in 2 mL of methanol under sonication. Then, 100 μL of 2,4-dimethylbenzenethiol was added and a yellow precipitate was formed immediately. After 5 minutes, 15 mL of dichloromethane was added, and the mixture was stirred for 30 minutes in an ice bath. Later, a freshly prepared PPh_4Br (7 mg in 0.5 mL methanol) solution was added before the drop-wise addition of ice-cold aqueous $NaBH_4$ (17 mg in 0.5 mL water). The yellow mixture turned to dark gradually. The stirring was stopped after 6 hours, and the mixture was allowed to age in a refrigerator for overnight. Then, the solvent was removed by rotary evaporation and the crude product was washed with methanol four times to remove the excess thiol and side products. Pure $Ag_{25}(2,4\text{-}DMBT)_{18}^{-}$ was extracted from the residue with acetonitrile. Crystals of $Ag_{25}(2,4\text{-}DMBT)_{18}^{-}$ were obtained by diffusion of hexane into a DCM solution of $Ag_{25}(2,4\text{-}DMBT)_{18}^{-}$.

Synthesis of $AuAg_{24}(2,4\text{-}DMBT)_{18}^{-}$: $AuAg_{24}(2,4\text{-}DMBT)_{18}^{-}$ was synthesized following a previously reported protocol with small modifications.[s5] First, 20 mg of $Ag_{25}(2,4\text{-}DMBT)_{18}^{-}$ was dissolved in 5 mL of DCM. Then, 1.6 mg of $AuClPPh_3$ was added to the solution. The mixture was slowly stirred for about 2 hours. The color of the solution gradually changed from dark reddish-brown to dark green. After the reaction, the mixture was centrifuged to remove AgCl, Ag-thiolates and any other insoluble side products. The clear DCM part was rotary evaporated and pure $AuAg_{24}(2,4\text{-}DMBT)_{18}^{-}$ was extracted from the residue with acetonitrile. Crystals of $AuAg_{24}(2,4\text{-}DMBT)_{18}^{-}$ were obtained by diffusion of hexane into a DCM solution of $AuAg_{24}(2,4\text{-}DMBT)_{18}^{-}$.

Synthesis of $Au_xAg_{25-x}(2,4\text{-}DMBT)_{18}^{-}$: $Au_xAg_{25-x}(2,4\text{-}DMBT)_{18}^{-}$ was synthesized by a reaction between $AuAg_{24}(2,4\text{-}DMBT)_{18}^{-}$ and Au(I)-(2,4-DMBT).[s6-7] To obtain Au(I)-(2,4-DMBT), 140 μL 2,4-dimethylbenzenethiol was added to a methanol solution containing 77 mg of $HAuCl_4{\cdot}3H_2O$. After 2 hours, the solvent was removed by rotary evaporation and the white product was washed by methanol for four times. To prepare $Au_xAg_{25-x}(2,4\text{-}DMBT)_{18}^{-}$, 20 mg of Au(I)-(2,4-DMBT) was added into a DCM solution (10 mL) of 10 mg $AuAg_{24}(2,4\text{-}DMBT)_{18}^{-}$ under stirring. The reaction was allowed to continue for 5 hours before the mixture was centrifuged to remove insoluable side products. Then, the solvent was removed and the as-obtained crude product was washed by methanol three times. Pure $Au_xAg_{25-x}(2,4\text{-}DMBT)_{18}^{-}$ was extracted from the residue with acetonitrile. Crystals of $Au_xAg_{25-x}(2,4\text{-}DMBT)_{18}^{-}$ were obtained by diffusion of hexane into a DCM solution of $Au_xAg_{25-x}(2,4\text{-}DMBT)_{18}^{-}$.

Preparation of NCs/polystyrene composite thin film. The drop-cast method was used to prepare the solid thin film. First, a stock solution of NCs was prepared by dissolving NCs (1 mg) in DCM (1 mL). Meanwhile, a polystyrene stock solution was prepared by dissolving polystyrene (80 mg) in DCM (1 mL). Then, 20 μL of as-obtained NC solution and 10 μL of polystyrene solution were mixed to afford an ink-like solution. Finally, the solution was dropped on a quartz plate, which was allowed to dry slowly at room temperature.

Steady-state UV-Vis-NIR absorption measurements. UV-Vis-NIR spectra of the nanoclusters were collected with a UV-3600 Plus UV-VIS-NIR spectrophotometer (Shimadzu, detection range: 185 to 3300 nm).

Steady-state photoluminescence and cryogenic measurements. Steady state photoluminescence spectra were measured on a FLS-1000 spectrofluorometer (Edinburgh). Near infrared PL was measured using a wide range InGaAs detector cooled by liquid nitrogen (-80 °C). The home-built low temperature PL system includes the FLS-1000 spectrofluorometer, a vacuum pump, an Optistat CF2 cryostat (Oxford Instruments) and a temperature controller. Liquid helium is used as cryogen.

Time-resolved photoluminescence measurements. The time-resolved PL measurements were carried out using a multi-channel scaling (MCS) single photon counting technique. An EPL-450 picosecond pulsed diode laser (Edinburgh Instruments) was used as the light source. The excitation wavelength from EPL-450 centered at 450 nm with a variance smaller than 5 nm. The pulse duration is less than 100 ps. For all MCS measurements, the detection wavelengths were set at the center of peaks.

Relative quantum yield calculation. The relative quantum yield (Φ_S) of the sample can be calculated using:

$$\Phi_S = \Phi_R\left(\frac{I_S}{I_R}\right)\left(\frac{1-10^{-A_R}}{1-10^{-A_S}}\right)\left(\frac{n_S}{n_R}\right)^2$$

where, Φ_R is the quantum yield of the reference standard, *I* is the integrated PL intensity, *A* is the absorbance of the solution at the excitation wavelength, and *n* is the refractive index of the solution solvent. (*S* and *R* represent sample and reference respectively.)

The absolute QYs were measured by using an integrating sphere (for details, see the note under Figure S7).

Supporting figures:

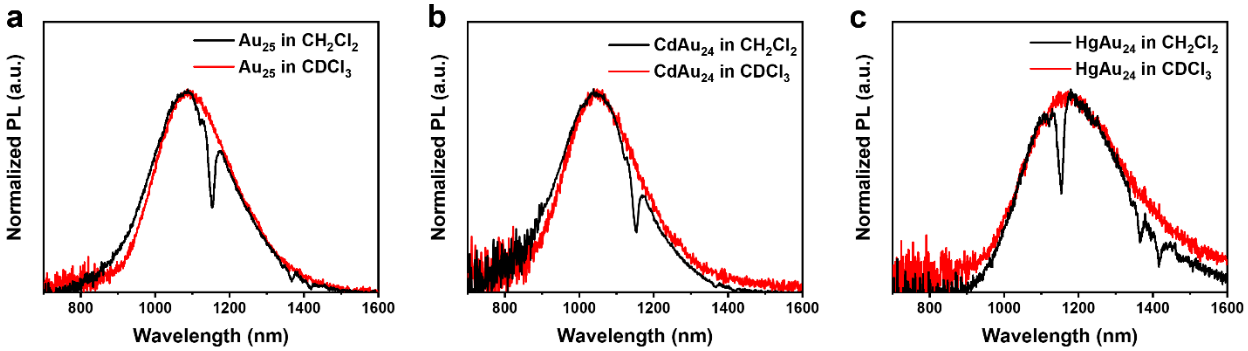

Figure S1. A comparison of PL spectra for (a) Au_{25}, (b) $CdAu_{24}$ and (c) $HgAu_{24}$ in DCM and deuterated chloroform.

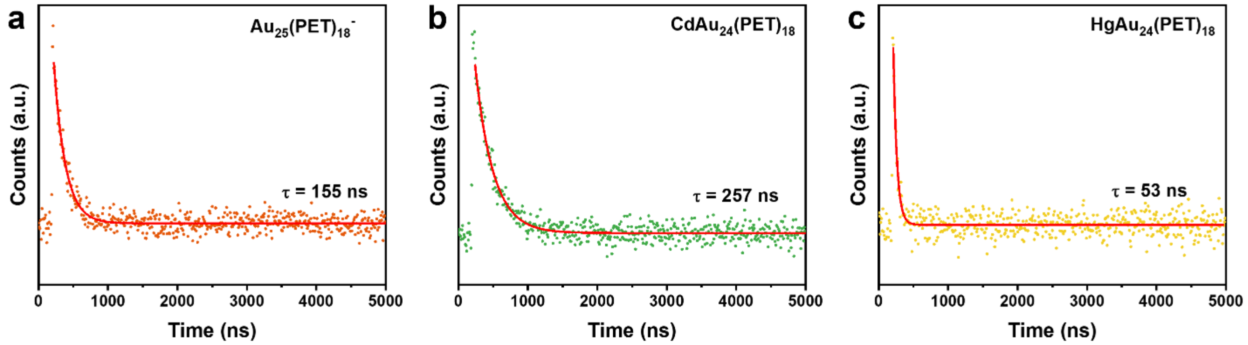

Figure S2. PL decay curves of (a) Au_{25}, (b) $CdAu_{24}$ and (c) $HgAu_{24}$ in deuterated chloroform. (Red lines are the fitting results using a mono exponential function.)

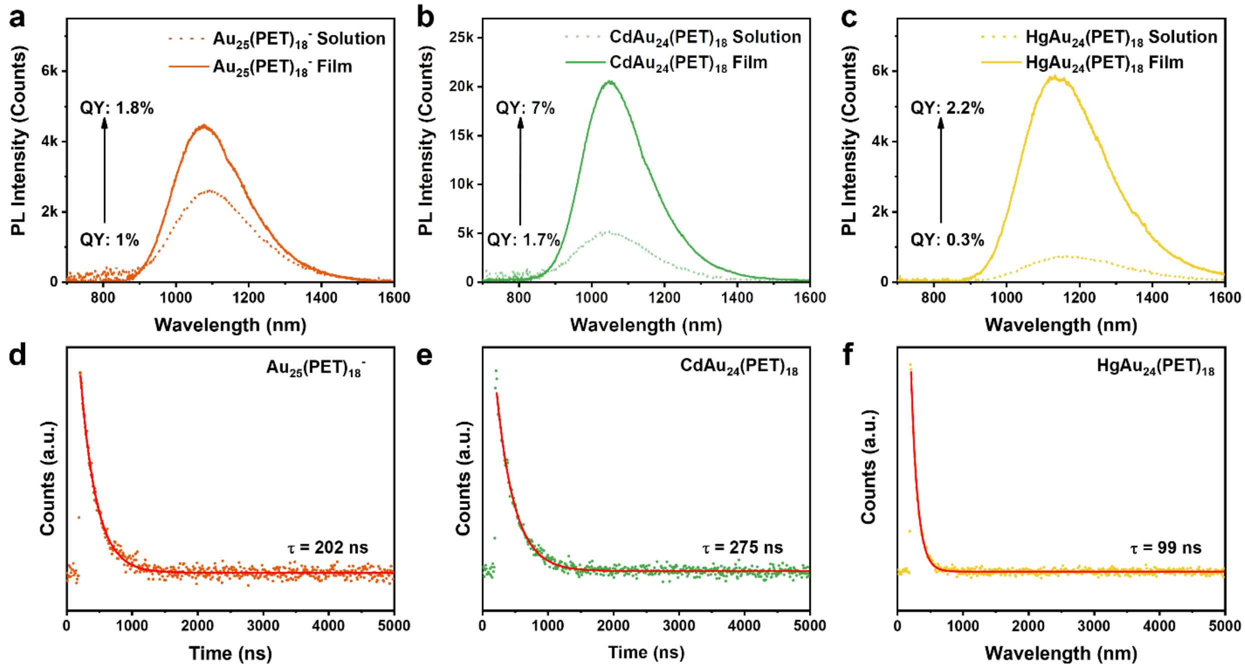

Figure S3. A comparison of PL spectra for (a) Au_{25}, (b) $CdAu_{24}$ and (c) $HgAu_{24}$ NCs in deuterated chloroform and polystyrene thin-film. Decay curves of (d) Au_{25}, (e) $CdAu_{24}$ and (f) $HgAu_{24}$ NCs in polystyrene thin-film. (Red lines are the fitting results using a mono exponential function.)

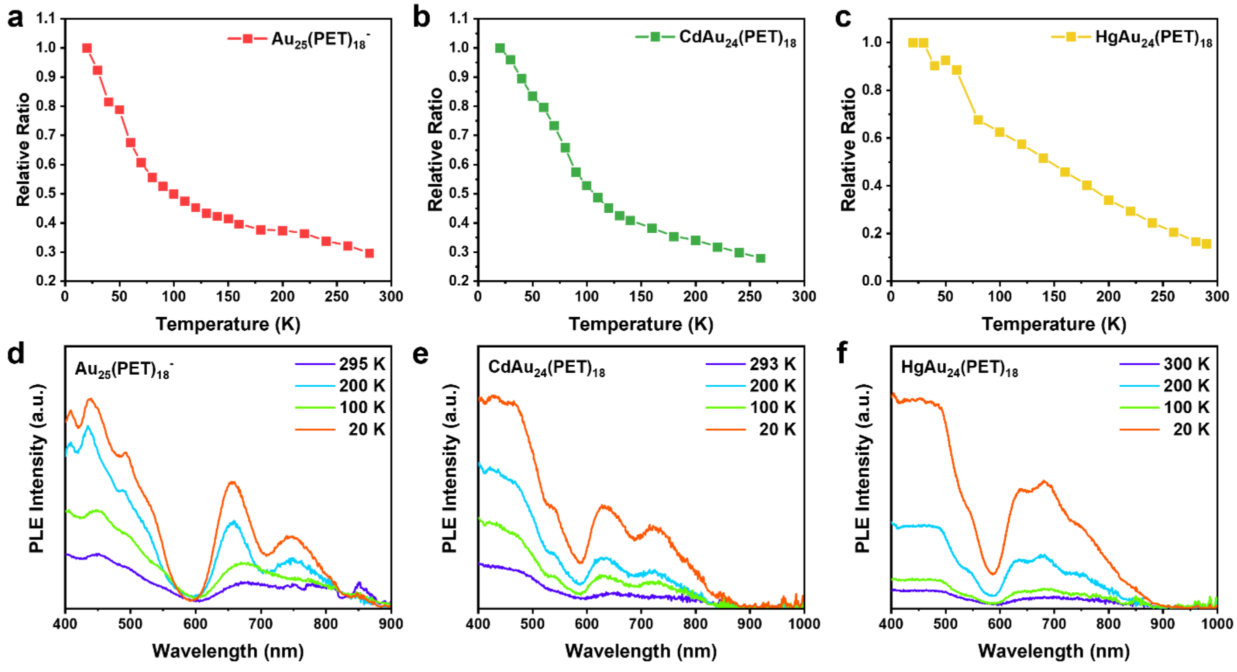

Figure S4. Normalized PL intensity of (a) Au_{25}, (b) $CdAu_{24}$ and (c) $HgAu_{24}$ at different temperatures. PL excitation spectra of (d) Au_{25}, (e) $CdAu_{24}$ and (f) $HgAu_{24}$ at different temperatures.

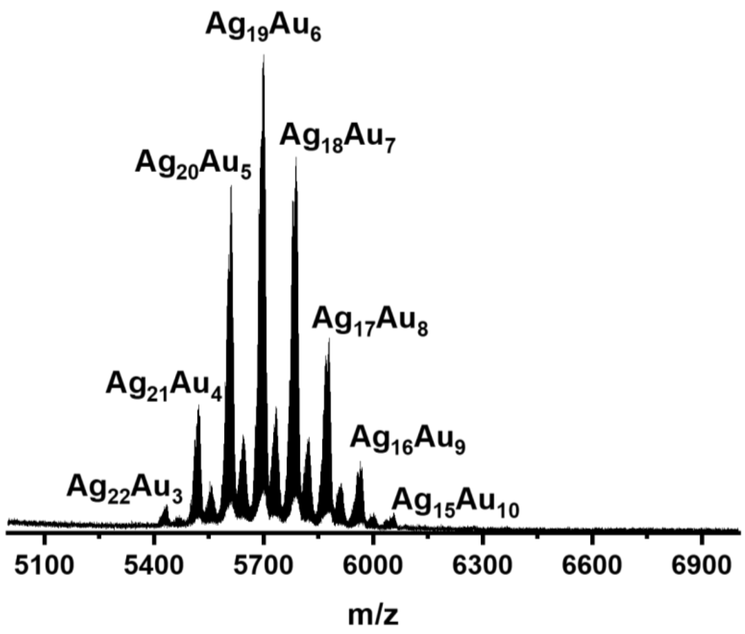

Figure S5. ESI-MS spectrum of $Au_xAg_{25-x}(2,4\text{-}DMBT)_{18}^-$ (x = 3 to 10).

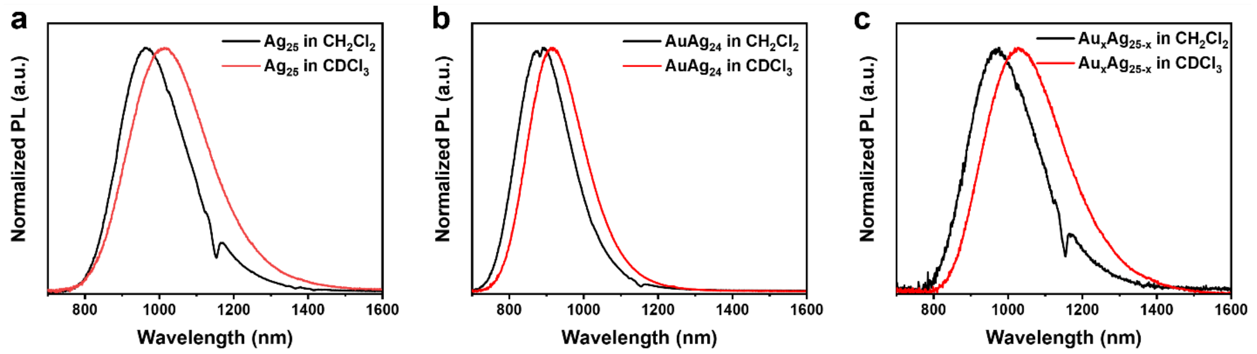

Figure S6. A comparison of PL spectra of (a) Ag_{25}, (b) $AuAg_{24}$ and (c) Au_xAg_{25-x} in DCM and deuterated chloroform.

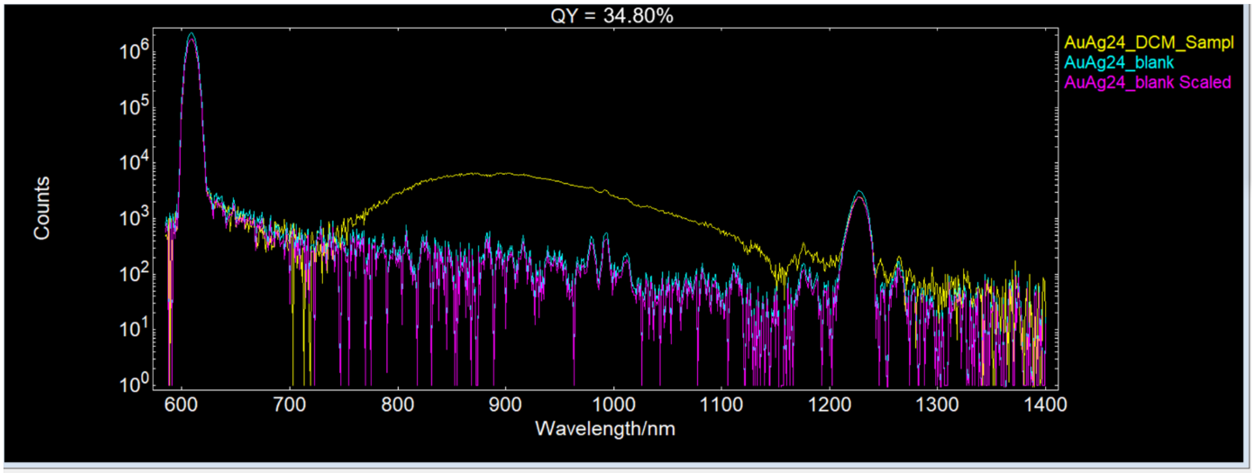

Figure S7. PLQY of $AuAg_{24}(2,4\text{-DMBT})_{18}^{-}$ in deaerated $CDCl_3$ at room temperature measured by an integrating sphere.

Note: For the absolute QY measurements, the normal sample holder is removed, and the integrating sphere is mounted in the FLS1000 spectrometer. The blank spectrum (blue line in Figure S7) was obtained by putting a blank into the sphere and collecting the emission scan (repeat 5 times) from 590 nm to 1400 nm with 610 nm excitation. The sample spectrum (yellow) was measured by replacing the blank with a dilute $AuAg_{24}$ solution (0.1 OD at 610 nm) and repeating the emission scan (5 scans) from 590 nm to 1400 nm with 610 nm excitation. The PLQY is calculated by:

$$\eta = \frac{L_{sample}}{E_{blank} - E_{sample}}$$

, where E_{blank} is the area under the blue line between 590 nm and 630 nm, E_{sample} is the area under the yellow line between 590 nm and 630 nm, L_{sample} is the area under the yellow line between 700 nm and 1200 nm.

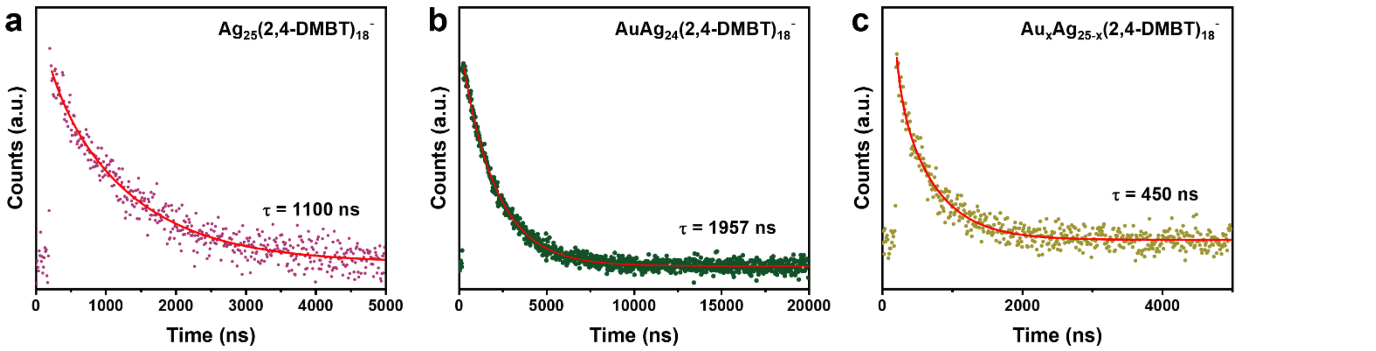

Figure S8. Decay curves for (a) Ag_{25}, (b) $AuAg_{24}$ and (c) Au_xAg_{25-x} in deuterated chloroform. (Red lines are the fitting results using a mono-exponential function.)

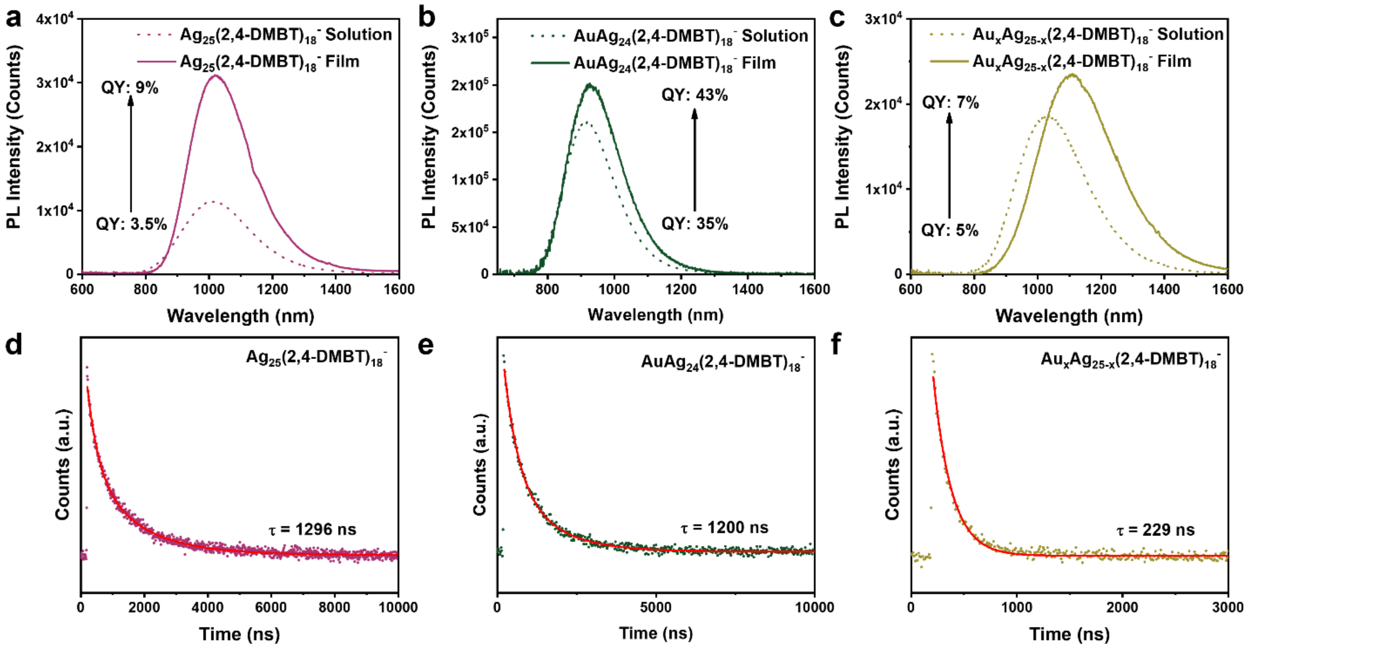

Figure S9. A comparison of PL spectra for (a) Ag_{25}, (b) $AuAg_{24}$ and (c) Au_xAg_{25-x} NCs in deuterated chloroform and polystyrene thin-film. Decay curves for (d) Ag_{25}, (e) $AuAg_{24}$ and (f) Au_xAg_{25-x} NCs in polystyrene thin-film. (Red lines are the fitting results using a mono exponential function.)

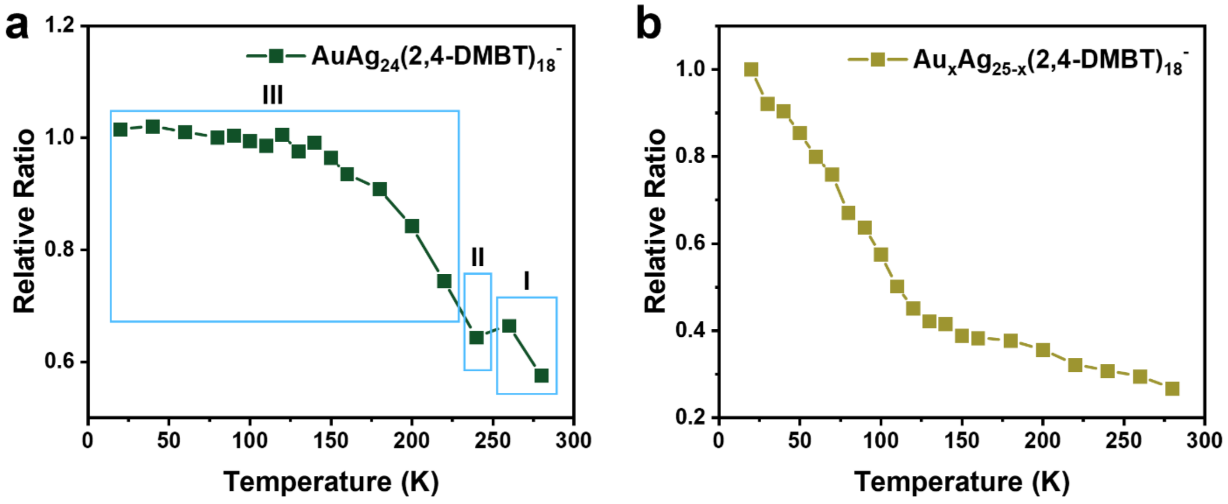

Figure S10. Normalized (integrated) PL intensity of (a) $AuAg_{24}$ and (b) Au_xAg_{25-x} at different temperatures.

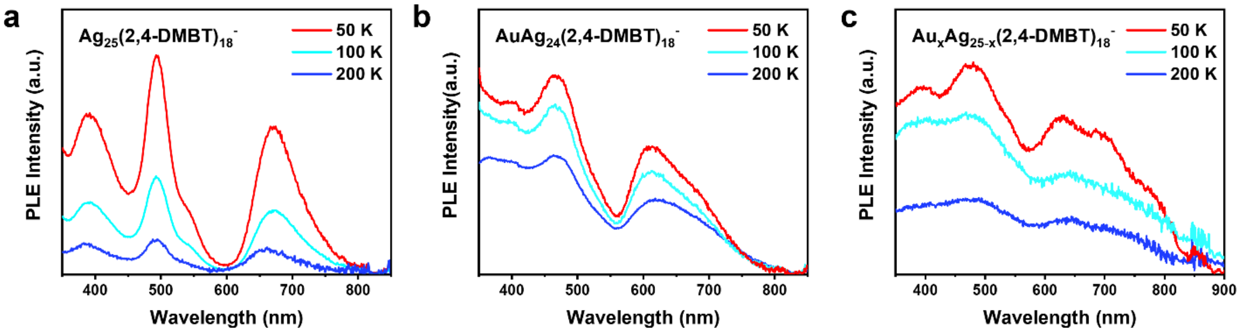

Figure S11. PL excitation spectra of (a) Ag_{25}, (b) $AuAg_{24}$ and (c) Au_xAg_{25-x} NCs at different temperatures.

Table S1. Temperature dependent PL lifetime of Au_{25}, $CdAu_{24}$ and $HgAu_{24}$.

	Lifetime of Au_{25} / ns	Lifetime of $CdAu_{24}$/ ns	Lifetime of $HgAu_{24}$/ ns
290	202	275	99
260	244	346	132
240	279	388	156
220	325	442	188
200	353	502	218
180	396	563	258
160	421	633	293
140	486	733	331
120	534	883	367
100	574	1024	399
80	627	1306	432
60	721	1585	567
40	805	2327	650
20	921	2817	720

Table S2. Temperature dependent PL lifetime of Ag_{25}, $AuAg_{24}$ and Au_xAg_{25-x}.

	Lifetime of Ag_{25} /ns	Lifetime of $AuAg_{24}$ /ns	Lifetime of Au_xAg_{25-x} /ns
280	1296	1200	229
260	1549	1323	205
240	2252	1523	238
220	3370	1821	281
200	4273	2239	322
180	5490	2665	411
160	9794	3192	413
140	12577	3958	531
120	19329	4568	616
100	21099	5134	815
80	24822	5486	811
60	>25000*	5750	1021
40	>25000*	5933	1248
20	>25000*	6132	1414

Note: * >25 μs, beyond the detector range.

Supporting refs:

(s1) Wu, Z.; Suhan, J.; Jin, R. One-Pot Synthesis of Atomically Monodisperse, Thiol-Functionalized Au_{25} Nanoclusters. *J. Mater. Chem.* **2009**, *19*, 622-626.

(s2) Fei, W.; Antonello, S.; Dainese, T.; Dolmella, A.; Lahtinen, M.; Rissanen, K.; Venzo, A.; Maran, F. Metal Doping of $Au_{25}(SR)_{18}^{-}$ Clusters: Insights and Hindsights. *J. Am. Chem. Soc.* **2019**, *141*, 16033-16045.

(s3) Suyama, M.; Takano, S.; Tsukuda, T. Synergistic Effects of Pt and Cd Codoping to Icosahedral Au_{13} Superatoms. *J. Phys. Chem. C* **2020**, *124*, 23923-23929.

(s4) Joshi, C. P.; Bootharaju, M. S.; Alhilaly, M. J.; Bakr, O. M. $[Ag_{25}(SR)_{18}]^{-}$: The “golden” silver nanoparticle. *J. Am. Chem. Soc.* **2015**, *137*, 11578-11581.

(s5) Bootharaju, M. S.; Joshi, C. P.; Parida, M. R.; Mohammed, O. F.; Bakr, O. M. Templated Atom-Precise Galvanic Synthesis and Structure Elucidation of a $[Ag_{24}Au(SR)_{18}]^{-}$ Nanocluster. *Angew. Chem.* **2016**, *128*, 934-938.

(s6) Pniakowska, A.; Kumaranchira Ramankutty, K.; Obstarczyk, P.; Perić Bakulić, M.; Sanader Maršić, Ž.; Bonačić-Koutecký, V.; Bürgi, T.; Olesiak-Bańska, J. Gold-Doping Effect on Two-Photon Absorption and Luminescence of Atomically Precise Silver Ligated Nanoclusters. *Angew. Chem. Int. Ed.* **2022**, *61*, e202209645.

(s7) Zheng, K.; Xie, J. Composition-Dependent Antimicrobial Ability of Full-Spectrum Au_xAg_{25-x} Alloy Nanoclusters. *ACS Nano* **2020**, *14*, 11533-11541.
